# Effect of *Cosmos Caudatus* supplementation and aerobic exercise on selected neurobehaviour, biochemical profile and histology in rats with mild cognitive impairment (MCI) induced by AlCl_3_: Study Protocol

**DOI:** 10.1371/journal.pone.0349933

**Published:** 2026-07-08

**Authors:** Daren Kumar Joseph, Arimi Fitri Mat Ludin, Farah Wahida Ibrahim, Nur Aishah Che Roos, Muhammad Hafiz Zuhdi Fairof, Suzana Shahar, Nor Fadilah Rajab

**Affiliations:** 1 Center for Healthy Ageing and Wellness (H-CARE), Faculty of Health Sciences, Universiti Kebangsaan Malaysia, Jalan Raja Muda Abdul Aziz, Kuala Lumpur, Wilayah Persekutuan Kuala Lumpur, Malaysia; 2 Center for Toxicology and Health Risk Studies (CORE), Faculty of Health Sciences, Universiti Kebangsaan Malaysia, Jalan Raja Muda Abdul Aziz, Kuala Lumpur, Wilayah Persekutuan Kuala Lumpur, Malaysia; 3 Faculty of Medicine and Defence Health, National Defence University of Malaysia, Kuala Lumpur, Malaysia; University of Rijeka Faculty of Health Studies: Sveuciliste u Rijeci Fakultet zdravstvenih studija, CROATIA

## Abstract

**Background:**

*Cosmos caudatus (C. caudatus)* or ‘ulam raja’ is a local plant with antioxidant properties and has the potential to act against oxidative-related conditions found such as in neurodegenerative diseases. Similarly, physical exercise is a consolidated strategy on the prevention of cognitive deficits. Based on the systematic review conducted by Joseph et al. (2023), a study protocol was developed to ensure the combined effect of *C. caudatus* supplementation and exercise provided improvement against cognitive impairment. There are limitations on studies looking at combined effect of flavonoid and exercise where either one of the interventions provided improvement to the behavioural tests and biomarkers assessed but not when given in combination. Moreover, to our understanding, in the last five years there has been limited research done on the combined effect of flavonoid and exercise against cognitive impairment (based on Pubmed search on 10 June 24; ScienceDirect search on 10 June 24). Therefore, we elucidated a study protocol that looks at the combined effect of *C. caudatus* supplementation and exercise against AlCl_3_-induced cognitive impairment in rats and the possible mechanisms involved in its neuroprotective effects in male rats.

**Method:**

Male Wistar rats will be divided into different groups: control, physical exercise (treadmill running), supplemented with *C. caudatus* or in combination. Consequently, neurobehavioural tests (novel object recognition test, open field test & Y-maze), biochemical tests and histology assessment will be determined to unravel the possible neuroprotective capability against AlCl_3_-induced neurotoxicity. The duration of exercise training is four weeks while *C. caudatus* is supplemented for 21 days.

**Outcome:**

The primary outcomes will be neurobehaviour changes at baseline, after 21 days of AlCl_3_-induced rats and at the end of intervention. While the secondary outcomes will be biochemical profile (Oxidative stress markers, inflammatory markers) and brain histology of AlCl_3_-induced rats.

**Discussion and conclusion:**

Combining exercise training with *C. caudatus* supplementation will produce synergistic effects, leading to significant improvements in spatial memory impairment and oxidative stress. This combined approach is expected to be more effective than using either intervention alone, potentially restoring spatial memory and antioxidant levels to normal. Consequently, the findings of this study could hold significant value for aging adults, providing safe and cost-effective strategies for managing neurodegenerative disorders.

## 1. Introduction

Mild cognitive impairment (MCI) represents a transitional stage between normal cognitive function and dementia [1]. MCI is categorized into two types: (1) amnestic MCI, characterized primarily by memory loss, and (2) non-amnestic MCI, where memory remains intact, but other cognitive abilities, such as organization, planning, reasoning, learning, or judgment, are impaired [2]. Cognitive decline imposes a significant economic burden on aging populations [3] and remains a global health and healthcare priority. Current pharmacological treatments for MCI provide modest improvements in cognitive function and neuropsychiatric symptoms for a limited period but do not offer a cure [4].

Nonsteroidal anti-inflammatory drugs (NSAIDs) are widely recognized for their anti-inflammatory and analgesic effects [5]. Long-term NSAID use has been associated with a reduced risk of dementia and slower onset and progression of Alzheimer’s disease (AD) [6]. NSAIDs may lower the risk of developing Alzheimer’s disease, but they do not slow its progression. This suggests that NSAIDs primarily target the initial pathological processes of the disease rather than the downstream mechanisms that drive its progression. Additionally, a prolonged period of administration may be necessary for NSAIDs to exert a protective effect. However, their use is limited by adverse side effects, particularly in patients with gastrointestinal ulcers, asthma, those on anticoagulant therapy, or individuals with kidney or liver disease, or during pregnancy [7]. As no current medications effectively treat MCI with minimal side effects, it is vital to explore non-pharmaceutical interventions to slow cognitive decline [8].

Exercise, defined as planned, structured, and repeated physical activity, aims to improve or maintain physical health and is crucial for healthy aging [9,10]. Regular exercise reduces the risk of chronic conditions such as coronary heart disease, stroke, type 2 diabetes mellitus, certain cancers, obesity, mental health issues, and neurological disorders like dementia [11]. Older adults who engage in regular exercise are more likely to maintain cognitive function compared to those who do not [12]. Aerobic exercise, in particular, enhances cognitive function and brain plasticity, showing positive outcomes in neurodegenerative diseases like Parkinson’s and Alzheimer’s [13,14]. Johansson et al. (2022) [15] found that aerobic exercise improved cognitive control and reduced global brain atrophy. Marques-Aleixo et al. [16] reported that physical exercise modulates brain structure and function, promoting a healthier neurological phenotype.

Dietary interventions also support cognitive health. Diets rich in vegetables, fruits, and fish enhance learning and memory [17]. Flavonoid-rich foods, such as berries, nuts, leafy greens, and citrus fruits, have demonstrated protective effects against neurodegenerative disorders [18–21]. Flavonoid-rich nutraceuticals have been used as food supplements to improve cognitive function and prevent neurodegenerative diseases in humans since ancient times [22]. Flavonoids are further divided into six classes based on their chemical structure: flavanols, flavanones, flavones, flavonols, flavonoids, and anthocyanidins [23]. In recent years, numerous indigenous herbs rich in polyphenols and antioxidants have shown beneficial effects on the cognitive function of older adults, including Persicaria minor aqueous extract [24], Ginkgo biloba [25,26], and Centella asiatica [27]. Another traditional plant that may help prevent neurodegenerative diseases is Cosmos caudatus (C. caudatus), due to its high total phenolic content and free radical scavenging properties [28]. C. caudatus, commonly known as Ulam raja in Malaysia and Kenikir in Indonesia, is a medicinal herb belonging to the Asteraceae family, which comprises 20–26 species worldwide [29]. Traditionally consumed as a salad, it has been widely recognized for its health benefits, including antihypertensive, antidiabetic, antioxidant, antiosteoporosis, antifungal, antibacterial, and antimutagenic properties [30,31,32]. Scientific studies have validated its potential for treating diseases linked to oxidative stress, such as cataracts, atherosclerosis, diabetes [33], Alzheimer’s [28], and other neurological disorders. C. caudatus demonstrates a high total antioxidant capacity, with proanthocyanidins, quercetin glycosides, and chlorogenic acids among its major bioactive compounds. Additionally, the plant contains phenolic compounds, flavonoids, flavones, and flavanones, which contribute to its ability to scavenge free radicals and prevent oxidative damage to tissues and cells. Its diverse bioactive properties underscore its potential for therapeutic applications [34]. According to a study by Ahda et al. [35], the pharmacological activities of C. caudatus largely depend on the type of extract used, as different solvents employed in the extraction process can result in variations in the bioactive metabolites obtained from the plant material. Notably, an aqueous extract of C. caudatus at a dose of 100 mg/kg administered for 21 days demonstrated significant antioxidant effects, highlighting its potential for therapeutic applications [36].

Emerging evidence supports the combined benefits of dietary intake and physical exercise as non-pharmacological strategies to improve cognitive impairment. This study aims to evaluate the synergistic effects of C. caudatus supplementation and aerobic exercise on neurobehaviour, biochemical profiles, and brain histology in AlCl_3_-induced MCI in rats. Aluminum (Al) is chosen since it is the third most abundant element on Earth and is widely encountered by humans through food, water, air, dust, and medicines [37]. Additional exposure arises from cooking or storing food in aluminum utensils and foils, as well as its use in industries such as paper production, water treatment, and pharmaceuticals [38]. Al is a recognized neurotoxic agent implicated in Alzheimer’s disease (AD). It has been detected in both senile plaques and neurofibrillary tangle (NFT)-bearing neurons in AD patients, suggesting its involvement in the disease [39]. Aluminum binding enhances the penetration of Aβ proteins across the blood-brain barrier and contributes to AD-like pathological changes, including tau and Aβ accumulation, neuronal apoptosis, and disruption of iron and calcium homeostasis [40,41]. Consequently, Al-induced AD models are widely used to screen potential anti-AD drugs. The primary mechanisms underlying Al-induced AD are linked to oxidative stress and autophagy dysfunction. Aluminum exposure has been shown to increase reactive oxygen species (ROS) production, induce lipid peroxidation, and impair antioxidant defenses by reducing the activity of enzymes like superoxide dismutase (SOD), catalase (CAT), and glutathione peroxidase (GPx) [42,43].

We will assess neurobehavioural changes at baseline (day 0 before induction of AlCl_3_), after induction and 2 days before supplementation of C. caudatus and/or exercise training and lastly at the end of intervention before sacrifice, alongside oxidative stress markers, inflammatory mediators, metabolomic profiles, and histological findings. Oxidative stress in the brain manifests as lipid peroxidation, indexed by elevated levels of thiobarbituric acid reactive substances (TBARS), and protein oxidation, indicated by protein carbonyl levels. Amyloid-β (Aβ), a major etiology of Alzheimer’s disease, causes an inflammatory response in the brain, leading to the production of multiple pro-inflammatory mediators such as inducible nitric oxide synthase (iNOS) and cyclooxygenase-2 (COX-2). Enzymes such as choline acetyltransferase (ChAT) and superoxide dismutase (SOD) activity are assessed in brain homogenate. In certain neurodegenerative diseases, a significant decrease in ChAT activity and concentration is detected, followed by neuronal loss. Additionally, quercetin, a bioactive compound found in C. caudatus, can decrease iNOS expression [44]. Brain-derived neurotrophic factor (BDNF), the most abundant growth factor in the central nervous system (CNS), plays a crucial role in hippocampal neurogenesis, synaptic plasticity, and learning and memory processes [45]. Evidence suggests that BDNF levels and metabolism in highly plastic brain regions, such as the cortex and hippocampus, are altered during normal aging and in Alzheimer’s disease [46]. For example, BDNF plasma levels are significantly reduced in elderly individuals, and BDNF expression is downregulated in the hippocampus of aged rodents [47,48]. MicroRNAs (miRNAs) are small, evolutionarily conserved, non-coding single-strand RNA molecules (18–25 nucleotides) that bind to the 3′ untranslated regions (3′ UTR) of target mRNAs, regulating their degradation or translation. Abundantly expressed in the nervous system in a tissue-specific manner, miRNAs play essential roles in neurogenesis, neuronal maturation, synapse formation, axon guidance, neurite outgrowth, and neuronal plasticity [49]. The role of microRNAs (miRNAs) in the development of Alzheimer’s disease has also been identified, with functional miRNAs considered potential therapeutic targets [50]. Acute endurance exercise increases the amount of SOD in the cerebral cortex of rats [51]. A substantial gene in anti-aging is Sestrin that helps regulate ROS and mechanistic target of rapamycin (mTOR) levels. These highly conserved proteins are activated under stress conditions such as radiation, hypoxia, starvation, DNA damage, and oxidative stress. Once activated, Sesn protects cells by managing signaling pathways critical for autophagy, growth, and cellular function [52].

Several studies have documented the amplification of nuclear factor kappa B (NF-κB) activity due to Aβ deposition, which is highly expressed in the brains of Alzheimer’s disease (AD) patients [53]. Additionally, Aβ overexpression has been shown to accelerate the degradation of acetylcholine (ACh), a neurotransmitter essential for cognition and memory [54]. Reactive oxygen species (ROS) play a critical role in activating various downstream signaling molecules, including mitogen-activated protein kinases (MAPKs). Among these, p38 mitogen-activated protein kinase (p38MAPK) has gained significant attention due to its response to diverse stress stimuli, such as ROS and inflammatory cytokines. Studies have linked p38MAPK to AD pathologies, suggesting that inhibiting the p38MAPK pathway could serve as a potential therapeutic target for AD [55,56]. Additionally, peroxisome proliferator-activated receptor gamma (PPAR-γ), a subtype of PPARs, is expressed in microglia and astrocytes, where it plays a key role in regulating inflammation and various CNS pathways [57,58]. Activation of PPAR-γ has been shown to reduce Aβ and tau accumulation, suppress neuroinflammation, and improve memory and learning [59,60].

In this study, we aim to investigate the potential interaction between *C. caudatus* supplementation and exercise training in AlCl_3_-induced rat model. The study is designed to explore whether their combined administration may demonstrate synergistic, agonistic, or antagonistic effects on neurobehavioural performance and biomarkers. These hypotheses are grounded in previous evidence suggesting individual benefits of both interventions. By examining their interaction within a controlled experimental framework, this protocol outlines a systematic approach to identify potential biomarkers relevant to neuroprotection and to inform future research on strategies that may contribute to healthier ageing and the prevention of neurodegenerative conditions.

### 1.1. Primary endpoint

Changes in biochemical profile of the following parameters:

- SOD (plasma & brain)
- ChAT activity
- Aβ1-42 level
- SOD level
- iNOS
- COX-2
- Sestrin + BDNF
- TBARS
- Protein Carbonyl

### 1.2. Secondary endpoint

Neurobehaviour changes will be assessed at baseline (before induction of AlCl_3_), after induction of AlCl_3_ and 2 days before supplementation of C. caudatus and/or exercise training and lastly at the end of intervention before animal sacrifice. The neurobehaviours tested are Open Field Test (OFT) which measures locomotor activity, Y-maze which measures spatial working memory and Novel Object Recognition Test (NORT) which measures learning and memory in rats.

## 2. Methodology

### 2.1. Experimental design

A total of 80 adult male Wistar rats (180 ± 20 g, age: six-eight weeks) will be obtained from the animal house Faculty of Medicine, Universiti Kebangsaan Malaysia (UKM). Animals will be maintained in a 12:12-h light-dark cycle (light on between 7:00 a.m. and 7:00 p.m.) at 23 ± 2°C and food and water will be available ad libitum. The rats will be chosen randomly. At first, eight cages will be selected and then the treatment procedure that will be written on pieces of paper will be randomly placed inside each cage. Next, the rats will be assigned one by one to different cages. Finally, animals will be divided into 5 sub-groups respectively (n = 16/group), see S1 Fig for experimental design. The animals in the same group will be housed on identical shelves in one room and treated daily for eight weeks. The animal handling and experimental procedures has been approved by ethics committee of UKM (FSK/2020/NOR FADILAH/23-SEPT./1133-SEP.-2020-FEB.-2023).

After one week of acclimatization, baseline neurobehaviour assessment will be conducted followed by induction of AlCl_3_/natural saline (Day 0) for eight weeks. Animals in the CC group will be administered C. caudatus extract after five weeks of AlCl_3_, for 21 days. While animals in the exercise group will begin treadmill exercise after four weeks of induction for a duration of 28 days. Cognitive impairment in rats will be determined by conducting neurobehaviour two days before C. caudatus administration and/or exercise training. For the animal grouping and intervention plan see S2 Fig.

Approximately 24 hours after the last neurobehaviour assessment, rats will be humanely euthanized. After decapitation, whole brain will be rapidly removed. From the 16 animals in each group brains will be processed as follows:

- Four animals will be sacrificed, and their whole brains will be rapidly removed for histopathological examination.
- Six animals will be used for biomarker analysis. The hippocampus homogenate will be prepared by extracting hippocampal tissues from brain samples stored at −80°C, which will then be rapidly sliced into small pieces on a cold plate.
- Six animals will be used for PCR analysis. The hippocampus will be rapidly dissected and snap-frozen using liquid nitrogen to preserve RNA integrity.

Whole brain is used for histology as the brain needs to be intact to avoid structural disruption as there will be loss of brain tissues during sectioning. This will ensure a clear view of the hippocampus area in histology assessment.

While for biomarker analysis homogenate of hippocampus will be prepared where the hippocampus from the brain samples kept at −80°C will be taken and rapidly sliced into small pieces on a cold plate. Additionally, the remaining will be kept at −40°C for further analysis. Brain samples will be homogenized in 0.1 M phosphate buffer (pH 7.4) to yield a 10% homogenate (w/v). The homogenate will be then centrifuged at 15000xg for 30 min at 40°C. The resulting supernatant obtained will be used for assaying Aβ peptide, protein carbonyl and Choline Acetyltransferase (ChAT), Inducible Nitric Oxide Synthase (iNOS), Cyclooxygenase-2 (COX-2), thiobarbituric acid-reactive substances (TBARS) assay and antioxidant enzymatic activities of Superoxide dismutase (SOD).

Trunk blood will be collected into sterile red-cap vacuum tubes with anticoagulant (EDTA). The samples will be centrifuged at 30,000xg for 15 minutes at 4°C and the plasma stored at −40°C for analysis of corticotropin-releasing hormone (CRH), Superoxide dismutase (SOD) and glutathione (GSH). See S3 Fig for timeline of animal study. As this article reports a study protocol, the experimental procedures, analytical approaches, and outcome measures described are intended for future implementation, and data collection has not yet started.

### 2.2. AlCl_3_ preparation

Aluminium Chloride (AlCl_3_) (20 mg/ml reagent grade, 98% (Sigma-Aldrich) will be dissolved in distilled water. AlCl_3_ is prepared weekly and administered daily.

### 2.3. Ibuprofen

Ibuprofen, extensively studied in clinical and experimental settings, has demonstrated benefits in addressing AD-related pathological factors, including reduced β-amyloid plaques, decreased amyloid precursor protein, and inhibition of Rho protein activity [61,62,63]. Nevertheless, Ibuprofen’s notable anti-inflammatory properties justify its use as a positive control in studies on MCI. Ibuprofen will be administered orally once per day for 4 weeks. In this study, a dosage of 17.5 mg/kg of ibuprofen was chosen to align with previous research as a positive control, representing a conventional anti-inflammatory medication [64].

### 2.4. *Cosmos caudatus* preparation and supplementation

The edible portions of fresh *Cosmos caudatus* were cleaned and washed under running tap water. The prepared plant material was then blended using an extractor. The resulting juice underwent filtration to separate the pure *C. caudatus* juice from the sediment, without the addition of water. The filtered juice was subsequently freeze-dried to produce a powdered extract. To maintain homogeneity, the *C. caudatus* samples were obtained from the same source. The plant material was purchased from the Institute of Bioproduct Development, Universiti Teknologi Malaysia (UTM) Skudai, located at the Skudai market. Liquid chromatography–mass spectrometry (LC–MS) analysis was conducted to identify the bioactive compounds present in *C. caudatus* prior to administration to the rats, ensuring that the observed outcomes were attributable to the active constituents of the plant.

For animal supplementation, *C. caudatus* extract was administered at a dose of 100 mg/kg body weight, dissolved in 0.1 mL distilled water, via oral gavage daily for 21 days. The dosage was based on previous findings, which showed that 100 mg/kg of *C. caudatus* aqueous extract had the potential to improve antioxidant biomarker levels after 21 days of treatment [36].

### 2.5. Experimental design timeline

A timeline of the experimental design is shown in S3 Appendix in S3 Fig. Adult male albino Wistar rats of six to eight weeks old of age will be obtained from the animal house Faculty of Medicine, *Universiti Kebangsaan Malaysia (UKM).* The animal handling and experimental procedures has been approved by ethics committee of UKM (FSK/2020/NOR FADILAH/23-SEPT./1133-SEP.-2020-FEB.-2023). Two rats will be housed in every standard polycarbonate cage in a temperature-controlled room at 23 ± 2°C under 12 h of light and 12 h of the dark cycle with free access to food and water ad libitum during the experiment. All rats will be allowed to undergo acclimatization for a week before the experiment.

After one week of acclimatization, baseline neurobehaviour assessment will be conducted followed by induction of AlCl_3_/natural saline (Day 0) for eight weeks. Animals in the CC group will be administered *C. caudatus* extract after five weeks of AlCl_3_, for 21 days. While animals in the exercise group will begin treadmill exercise after four weeks of induction for a duration of 28 days. Cognitive impairment in rats will be determined by conducting neurobehaviour two days before *C. caudatus* administration and/or exercise training. Approximately 24 hours after the last neurobehaviour assessment, rats will be humanely euthanized. After decapitation, whole brain will be rapidly removed.

### 2.6. Behavioural test

In this study, three behavioural tests will be conducted, namely open field test, novel object recognition task and Y-maze at baseline, after induction of AlCl_3_, 21/28 days of supplementation and/or training. All tests are selected because the techniques are easy to understand and the duration of the study is brief compared to other behavioural tests. In addition, the results obtained are also reliable as per the previous study [65].

#### 2.6.1. Open field test (OFT).

To measure locomotor activity in rats, rats will be placed in the open field arena before the novel object recognition task. Open field test aims to investigate exploration of novel environment, general locomotor activity and conduct early screening of behaviour related to anxiety in the rodents [66]. Experiments will be conducted in sound proofed rooms with a red fluorescence lighting source exceeding 20 watts so that rats in the arena could be recorded clearly. Each rat will be placed in the middle of the arena measuring 72 cm x 72 cm x 38 cm and the activity will be recorded for 5 minutes for two consecutive days to study the habituation process. The arena will be wiped with 70% Ethanol prior to use and before subsequent tests to remove any scent clues left by the previous rats. Ethanol must be allowed to evaporate completely prior to testing rats. This may take 5–10 min between each testing session. Recording of rats’ videos will be analysed and rats’ behaviours will be given score such as line cross, rearing, static frequency (freezing) and duration of admission into zone A (periphery), zone B (mid) and zone C (central square). Furthermore, the total number of locomotor activity per rat will be calculated based on the number of border crossing frequencies and the number of rears.

#### 2.6.2. Novel object recognition task (NORT).

The NORT will be conducted after the completion of OFT using the same arena, which will be divided into three phases namely the habituation phase, familiarization and novel [67]. Each rat will be first allowed to undergo a self-adjusting process (45 minutes in the test room and 5 minutes for an empty field exploration) for two consecutive days. Then, rats will be given two samples of the same shape and size for the familiarization process for 15 minutes. Exploration of objects will be given scores from camera video recording when a rat’s nose is within 1 cm of the object and the vibrissae (e.g., rat) will be seen moving. The rats will be removed and arena cleaned before this process is repeated and then one of the objects will be replaced by the novel object. In this novel phase, rats will be exposed with a novel object and a familiar object for 10 minutes to identify the memory location of the rats. The likelihood of rats to choose novel objects will be expressed as the percentage of time exploring novel objects by rats rather than familiar objects.

#### 2.6.3. Y-maze.

The Y-maze is a hippocampal dependent–spatial working memory task that requires rats to use external maze cues to navigate the identical internal arms. The Y-maze was chosen to reduce habituation time and provide a measure of spatial working memory and to limit stressful confounds such as food deprivation (radial arm maze) or forced swimming (water maze). The apparatus consists of a black plastic maze with three arms (50 cm long, 32 cm high and 16 cm wide) that is intersected at 120°. The plastic makes it easy to disinfect between animals to remove any odors. A rat will be placed at the end of one arm and allowed to move freely through the maze for 6 min without reinforcements, such as food and water. Entries into all arms will be noted (4 paws had to be inside the arm for a valid entry) and a spontaneous alternation will be counted if an animal entered three different arms consecutively.

### 2.7. Biomarkers analysis

#### 2.7.1. Real-time PCR (ChAT).

Quantitative real-time RT-PCR is performed to measure the related gene expression of insulin in ChAT. All real-time PCR reactions will be run in duplicate in Rotor-Gene Q thermocycler. The PCR master mix in each well contained 10 µl of RT2 SYBR Green ROX qPCR Master Mix, 7 µl dH2O, 1 µl mixture of the forward and reverse primers (10 pMeach), and 2 µl of single-stranded cDNA in a final reaction of 20 µl. The conditions for PCR amplifications will be as follows: initial denaturation and Taq DNA polymerase activation at 95°C for 10 min, and 40 cycles of 95°C for 10 s, 64°C for 60 s and 72°C for 60s. The percentage of changes to control group will be evaluated by the comparative Ct method [68].

#### 2.7.2. Brain and plasma superoxide dismutase (SOD) activity.

SOD level is measured by the ELISA method using the commercial ELISA kit (Cat.No.:E-EL-R1424, Elabscience^@^ Houston, TX, USA). The standard concentrations of ELISA kits will be as follows: 4000, 2000, 1000, 500, 250, 125, 62.5, 0 pg/ml. The optical density (OD) will be measured spectrophotometrically at a wavelength of 450nm. The concentration of SOD in the sample will be determined by comparing the OD of the samples to the standard curve.

#### 2.7.3. Inducible nitric oxide synthase (iNOS).

The iNOS level is measured by the ELISA method using the commercial ELISA kit (Cat.No.:E-EL-R0520, Elabscience^@^ Houston, TX, USA). The standard concentrations of ELISA kits will be as follows: 4000, 2000, 1000, 500, 250, 125, 62.5, 0 pg/ml. The optical density (OD) will be measured spectrophotometrically at a wavelength of 450nm.

#### 2.7.4. Cyclooxygenase-2 (COX-2).

The COX-2 level is measured by the ELISA method using the commercial ELISA kit (Cat.No.:E-EL-R0792, Elabscience^@^ Houston, TX, USA). The standard concentrations of ELISA kits will be as follows: 20, 10, 5, 2.5, 1.25, 0.625, 0.313, 0 ng/ml. The optical density (OD) will be measured spectrophotometrically at a wavelength of 450nm.

#### 2.7.5. Quantification of Aβ_1–42_.

Aβ will be extracted from brain tissues as described before [69]. The homogenates will be kept at 4°C for 15 min and then centrifuged at 100,000 g for 1 h. The formic acid extract layer between a thin overlying lipid layer and a small pellet will be removed and used for Aβ_1–42_ quantification by a Aβ_1–42_ ELISA kit (Cat.No.:E-EL-R1402, Elabscience^@^ Houston, TX, USA). The level of the Aβ_1–42_ in each brain sample will be standardized to the brain tissue weight. The results from animals under various experimental conditions will be then normalized by the mean values of the corresponding control animals in each ELISA assay.

#### 2.7.6. Plasma levels of corticotropin-releasing hormone (CRH).

One milliliter of plasma sample will be mixed with 2 ml of 6 M guanidine hydrochloride (Sigma-Aldrich, 50950), and the mixture will be applied to a SEP-PAK C18 cartridge (Waters, Massachusetts, USA). The column will be washed first with 10 ml of 0.1 N Hydrochloric acid (HCI) (Sigma-Aldrich, CAS No.:7647-01-0), then with distilled water. CRH will be eluted with 2.5 ml of a mixture of Acetonitrile (CH_3_CN) and 0.5% acetic acid (6:4, vol/ vol). The eluate will be lyophilized [70].

#### 2.7.7. Estimation of a lipid peroxidation assay (TBARS).

The assay for lipid peroxidation will be carried out following the modified method of Iqbal et al. [71]. The reaction mixture in a total volume of 1.0 ml contained 0.58 ml phosphate buffer (0.1 mol; pH 7.4), 0.2 ml homogenate sample, 0.2 ml ascorbic acid (100 mmol) (CAS No.:50-81-7), and 0.02 ml ferric chloride (100 mmol) (CAS No.:10025-77-1). The reaction mixture will be incubated at 37°C in a shaking water bath for 1 h. The reaction will be stopped by addition of 1.0 ml 10% trichloroacetic acid (CAS No.:76-03-9). Following addition of 1.0 ml 0.67% thiobarbituric acid (CAS No.:74669-22-0), all the tubes will be placed in boiling-water bath for 20 min and then shifted to crushed ice-bath before centrifuging at 2500 × g for 10 min. The amount of TBARS formed in each of the samples will be assessed by measuring optical density of the supernatant at 535 nm using a spectrophotometer against a reagent blank. The results will be expressed as nmol TBARS/min/mg tissue at 37°C using a molar extinction coefficient of 1.56 × 105 M-1 cm-1. Chemicals will be purchased from Merck KGaA, Darmstadt, Germany.

#### 2.7.8. Determination of protein carbonyl levels.

The homogenized tissue will be transferred to a plastic tube, left for 15 min at room temperature, and then streptomycin sulfate solution (10% w/v) was added to a final concentration of 1% to precipitate any extracted DNA which could react with DNPH (2,4-dinitrophenylhydrazine) (CAS No.:119-26-6) and contribute to the carbonyl level. The solution will be mixed and left to stand a further 15 min at room temperature, after which it will be centrifuged at 2800g for 10 min at room temperature. The supernatant will be removed and divided equally between two 10 ml plastic centrifuge tubes with the remaining supernatant being reserved for other assays. DNPH (1.6 ml, 10 mM in 2 M HCl) will be added to one tube and 1.6 ml of 2 M HCl to the other tube (ratio of supernatant to DNPH solution should be 1:4, v/v). The tubes will be then incubated for 1 h on a rotator at room temperature and then the protein will be precipitated by adding an equal volume of 20% (w/v) trichloroacetic acid (TCA) to the tubes and leaving them for 15 min. The protein will be spun down at 3400g (10 min, room temperature), the supernatant will be discarded, and the pellet will be washed with 1.5 ml of an ethyl acetate: ethanol mixture (1:1, v/v) to remove excess DNPH. This procedure will be repeated three times. The final protein pellet will be dissolved in 1.25 ml of 6 M guanidine hydrochloride and the absorbances of both solutions (DNPH and HCl) will be measured at 370 nm from which the PCO content could be evaluated (PCO concentration in nmol/ml: ΔA370 × 45.45, where ΔA370 equals A370 of DNPH solution−A370 of HCl solution). The protein concentration will be calculated from the A280 of the HCl samples (A280 × 1.8 gives protein concentration in mg/ml) [72].

#### 2.7.9. Measurement of PPAR-γ, p38MAPK, and NF-κB/p65 levels.

The status of PPAR-γ (Cat.No.:E-EL-R0724, Elabscience@ Houston, TX, USA), p38 MAPK (Catalog # 85-86022-11, Thermofisher Scientific, USA) and NF-κB/p65 (Cat.No.:E-EL-R0674, E lab Science) in the brain tissues of control and treated animals will be quantified using respective assay kits as the manufacturer’s protocols. The sequence for primers used in qRT-PCR is provided see S1 Table and the protocol is briefly described below:

**PPAR-γ:** Standards or samples will be added to the micro ELISA plate wells and combined with the specific antibody. Then a biotinylated detection antibody specific for Rat PPAR-γ and Avidin-Horseradish Peroxidase (HRP) conjugate will be added successively to each micro plate well and incubated. Free components will be washed away. The substrate solution will be added to each well. Only those wells that contain Rat PPAR-γ, biotinylated detection antibody and Avidin-HRP conjugate will appear blue in color. The enzyme-substrate reaction will be terminated by the addition of stop solution and the color turns yellow. The optical density (OD) is measured spectrophotometrically at a wavelength of 450 nm ± 2 nm. The OD value is expected to be proportional to the concentration of Rat PPAR-γ. The concentration of Rat PPAR-γ in the samples can be calculate by comparing the OD of the samples to the standard curve.

**p38 MAPK:** The InstantOne ELISA™ assay workflow begins with preparing the sample lysate, 50 µL of the sample lysate or 50 µL of lysis mix (Negative Control), or 50 µL of Control Lysate (Positive Control) will be added to the InstantOne™ ELISA microwell wells. Following this, 50 µL of freshly prepared antibody cocktail is added to each well, and the plate is incubated for 1 hour at room temperature while shaking at 350 rpm (remove the reagent at 4°C and let warm to room temperature). The plate will then washed three times using 200 µL of wash buffer per well. Subsequently, 100 µL of Detection Reagent will be added to each assay well, and the plate is incubated for 10–30 minutes while shaking at 300 rpm. Finally, 100 µL of Stop Solution will be added, and the absorbance is immediately read on a microplate reader set to 450 nm.

**NF-κB/p65:** This ELISA kit uses the Sandwich-ELISA principle. The micro ELISA plate provided in this kit has been pre-coated with an antibody specific to Rat NFKB-p65. Standards or samples will be added to the micro ELISA plate wells and combined with the specific antibody. Then a biotinylated detection antibody specific for Rat NFKB-p65 and Avidin-Horseradish Peroxidase (HRP) conjugate will be added successively to each micro plate well and incubated. Free components will be washed away. The substrate solution will be added to each well. Only those wells that contain Rat NFKB-p65, biotinylated detection antibody and Avidin-HRP conjugate will appear blue in color. The enzyme-substrate reaction will be terminated by the addition of stop solution and the color turns yellow. The optical density (OD) is measured spectrophotometrically at a wavelength of 450 nm ± 2 nm.

#### 2.7.10. RNA extraction and real-time polymerase chain reaction.

Total RNA will be extracted from the hippocampus using miRCURY™ RNA isolation kit (Qiagen, Doncaster, VIC, Australia) according to the manufacturer’s protocol. The RNA content and purity will be measured using the NanoDrop 1000 spectrophotometer. The expression profiles of miR-146a and miR-155 will be performed on total RNA extracted using the Universal cDNA Synthesis Kit. Briefly, total RNA containing miRNA is polyadenylated and cDNA will be synthesized using a poly (T) primer with a 30 degenerate anchor and a 50 universal tag. RevertAid First Strand cDNA Synthesis Kit (with the aid of random hexamer primers and MMLV reverse transcriptase (as a complete system for efficient synthesis of first-strand cDNA from mRNA or total RNA templates) will be used for determination of NF-ΚB mRNA expression levels. Real-time polymerase chain reaction reactions will be performed on a Bio-Rad iQ5 Detection System (Bio-Rad, Richmond, CA, USA). The 2^−(ΔΔCt)^ method is used to determine the relative-quantitative levels of individual mRNAs and miRs. The results will be expressed as the fold-difference to the relevant controls.

## 3. Treadmill exercise protocol

The 4-week treadmill exercise training protocol was designed based on previous findings which demonstrated that such training could ameliorate spatial learning and memory impairments [73] by regulating neuroinflammation and BDNF expression, thereby improving cognitive function [74]. The rats in the exercise groups will be made to run on the treadmill 30 min once a day, five times a week for 4 weeks, starting 4 weeks after AlCl_3_ administration, see S4 Fig. The workload of the exercise consisted of running at a speed of 3 meters/min for the first 5 min, 5 meters/min for the next 5 min, and then 8 meters/min for the last 20 min, with 0% grade of inclination. The animals in the non-treatment group and in the AlCl_3_ induced group will be placed in a similar setting for the same duration without running.

The exercise training will be conducted in the same environment where the animals are housed; therefore, acclimatization to the training setting is unnecessary. Exercise will be conducted in the evening from 6:00 to 10:00 PM, when the animals are most active. During the training sessions, three rats will be placed on the treadmill simultaneously. The treadmill is equipped with shock grids to motivate non-compliant rats to run. The working hypothesis is that treadmill running exercise will prevent aged-related memory deficits, and that such effect is possibly associated to a reduction of oxidative stress and to increased expression of neurotrophic factors in the hippocampus of aged rats. Previous study in Wistar rats showed treadmill running over 4 weeks, increased expression of BDNF in the hippocampus. An effect possibly associated to the reduction of oxidative stress [75].

## 4. Histology assessment

### I. Preserving brain tissue

The brain will be immediately immersed in 10% buffered formalin fixative for 48 h. This fixation process will be performed to maintain the structure of the tissue originally, to prevent tissue autolysis and to protect the tissues.

### II. Tissue processing

Dehydration is a process in which the tissue is immersed in a series of alcoholic concentrations upwards of 50%, 70%, 80%, 90%, and 100% (I and II). The immersion time for each concentration of alcohol is for 1 ½ hours except for alcohol I and II, the immersion time is 2 hours. For cleaning purposes, tissue will be immersed into xylene I and II. The soaking time for each is for 2 hours. The tissue impregnation process is performed three times where the tissue will be placed into paraffin wax I, II and III.

### III. Embedding the tissue

This process is done by using a tissue embedding machine. Paraffin wax will be inserted into a block-shaped mold and tissue embedded into the mold. Then, the tapes will be placed on the mold and paraffin will be filled up. The mold is labelled and transferred to the machine side which is 4^o^C so that the paraffin freezes. Once the paraffins freeze, the block will be removed from the mold and stored at 4^o^C.

### IV. Tissues and fishing processes (fishing)

Blended tissue blocks cool sliced with a microtome at a thickness of 20 µm to 50 µm. The ribbed tissue will then be transferred to a 60°C water bath. The ribs will be left floating on the surface horizontally. By using fishing techniques, tissue slices will be attached to a glass slide and transferred to a 60°C heating plate and left overnight.

### V. The process of creation and rehabilitation

This process will be performed by soaking the sliced tissue slide overnight into xylene I and II for 10 minutes for each immersion, followed by a rehydration process with a series of decreased alcohol concentration ranging from: 100% twice during 5 minutes each, 95% for 3 minutes, and 70% for 3 minutes.

### VI. Hematoxilin and Eosin (H & E) and dehydration staining

This tinting will be done after the rehydration process where the slab is immersed in hematoxyline for 10–15 minutes before flushing under running water for a while. After that, the slides will be immersed in a solution of 1% alcoholic acid and then rinsed under the running water for a while. Afterwards, the slides are immersed for 5–7 minutes in eosin solution followed by dehydration processes involving 95% (I and II) and 100% (I and II) alcohols for three minutes each. The last process is the cleaning process by immersing the slide in xylene for five minutes each as much as two copies. Lastly, the tissue will be covered with a glass lid using DPX and drops a little xylene to prevent foam on the slide.

### VII. Nissl coloring and dehydration

This method is used to detect Nissl’s body in the cytoplasm of the neuron in tissue cuts such as the part of the brain [76]. First, immerse the brain tissue in the xylene twice as long as 5 minutes for each solution. The rehydration process will be carried out in 95% and 70% alcohol for 3 minutes, followed by immersion in the dissolved water for 3 minutes. Next the slide will be rinsed with tap water and followed by distilled water. Then, the slide will be immersed in a cresyl violet dye solution heated at 37°C to 50°C in the oven for 30 minutes. This heating is done to improve absorption and improve the coloration quality evenly. Next, the slide will be rinsed with distilled water for 3 minutes before proceeding with dehydration by alcohol 70% (3 minutes), alcohol 95% (1–2 minutes) and 100% alcohol. The last process is the clearing process by immersing the slides in xylene for 5 minutes twice. Covered tissues will be closed to protect the tissue. DPX and xylene will be dropped above the tissue and then the glass inserts will be used to cover the tissues slowly to prevent air bubbles from forming.

### VIII. Calculation of cell neuron counts

Neuronal, glial and endothelial cells will be identified through cross-sections in slides that are colored with Nissl based on morphological criteria [77]. Neurons are distinguished from glia by size, the presence of euchromatin in the nucleus, the visible nucleolus with the cytoplasm around it. Glial cells are seen to have heterochromatin in the nucleus without clear cytoplasm. While the endothelial cell nucleus is part of a curved-shaped blood vessel. Since the glial and endothelial cell nucleolus cannot be seen clearly in all cases, therefore the nucleus of the nucleus is considered a unit number with the aid of an analysis software Image J. The frame area of the optical dissector is 10m x 10m = 100m^2^ with a small scapel, techniques from Akdogan, Unal.

### IX. Immunofluorescence staining

The cryoprotected sections will be washed with 0.1 M PBS three times for 5 min then incubated in 0.3% Triton X-100 in 0.1 M PBS for half an hour at room temperature. They will then be washed with 0.1 M PBS for 5 min, three times and incubated with primary antibodies: sestrin2, cleaved caspase3, LC3 A/B, DYDDDDK Tag, NeuN, respectively (4°C, overnight). After washing with 0.1 M PBS (5 min, three times), the slides will be incubated with secondary antibodies which are from Santa Cruz Biotechnology: anti-rabbit IgG-TR, anti-mouse IgG-FITC, anti-goat IgG-FITC, anti-rabbit IgG-FITC (1:200) for 1 h under room temperature, then washed again with 0.1 M PBS for 5 min, three times. Finally, slides will be covered with DAPI (Vector Laboratories, Inc.). Fluorescent microscope and Magna Fire SP system (Olympus) will be used to analyze microphotographs.

## 5. Treadmill exercise protocol

The rats in the exercise groups will be made to run on the treadmill 30 min once a day, five times a week for 4 weeks, starting 4 weeks after AlCl_3_ administration. The workload of the exercise consisted of running at a speed of 3 meters/min for the first 5 min, 5 meters/min for the next 5 min, and then 8 meters/min for the last 20 min, with 0% grade of inclination. The animals in the non-treatment group and in the AlCl_3_ induced group will be placed in a similar setting for the same duration without running.

The working hypothesis is that treadmill running exercise will prevent aged-related memory deficits, and that such effect is possibly associated to a reduction of oxidative stress and to increased expression of neurotrophic factors in the hippocampus of aged rats. Previous study in Wistar rats showed treadmill running over 4 weeks, increased expression of BDNF in the hippocampus. An effect possibly associated to the reduction of oxidative stress [75].

## 6. Statistical analysis

Once data collection has been completed, neurobehaviour changes will be analysed using mixed design ANOVA. Also, Tukey’s post-hoc test was used to compare significant differences. Furthermore, one-way ANOVA was used for assessing biochemical parameters and the statistical significance was determined by Tukey’s post-hoc test. P < 0.05 was regarded as statistically significant, and the results were expressed as mean ± standard deviation. Analysed data will be deposited in GIN – Modern Research Data Management for Neuroscience (https://gin.g-node.org/) under user DarenK.

## 7. Discussion

In order to delay the progression of MCI to dementia and Alzheimer’s disease (AD), early control and management of MCI are crucial [78]. Numerous non-pharmacological interventions have been examined, such as dietary adjustments, exercise, and cognitive stimulation [79].

Animal studies have consistently reported that physically active mice exhibit improved neuroplasticity and anti-inflammatory effects [80]. Physical exercise enhances learning, memory, and brain plasticity by promoting angiogenesis, neurogenesis, and synaptogenesis. Brain-derived neurotrophic factor (BDNF), the most abundant neurotrophin, is synthesized in peripheral tissues such as muscle, liver, adipose tissue, endothelial cells, and immune cells. Compared to resting conditions, the serum level of BDNF increases with aerobic exercise, which is linked to enhanced plasticity [81,82,83]. The accumulation of amyloid-beta (Aβ) induces oxidative stress in the brain, leading to lipid peroxidation, protein oxidation, and cognitive impairment. Regular physical activity helps regulate the oxidant/antioxidant balance [84]. A study by Jahangiri et al. [85] showed that moderate exercise decreased lipid peroxidation and increased the activity of superoxide dismutase (SOD) and catalase (CAT) in hippocampal tissues. Specifically, treadmill exercise has been shown to improve cognitive impairment by enhancing the oxidative state of the hippocampus in rats [86]. Furthermore, physical exercise effectively inhibits NF-κB activation and suppresses COX-2 expression, which plays a crucial role in the neuroinflammatory response [87,88].

In studies involving experimental animals and cell cultures, flavonoids have demonstrated neuroprotective properties and the ability to slow brain aging and cognitive decline. They achieve this by producing neuronal and inducible nitric oxide, which suppresses microglia-mediated inflammation, reduces blood pressure, and lowers oxidative stress, thereby reducing vascular risk [89]. Flavonoids can increase the activity of glutathione peroxidase (GSH-Px) and SOD and neutralize free radicals to enhance ATPase activity in rat brains [90]. They also alleviate behavioural and cognitive deficits due to their antioxidant potential by exhibiting acetylcholinesterase (AChE) inhibitory effects, which bolster the antioxidant defense mechanism of cholinergic neurons [91]. Additionally, flavonoids may prevent neuroinflammation by inhibiting inducible nitric oxide synthase (iNOS) induction and downregulating pro-inflammatory transcription factors such as NF-κB [92]. *C. caudatus* supplements are rich in flavonoids such as quercetin, catechin, epicatechin, and proanthocyanidins [31], and their antioxidant effects, such as those of quercetin, potentially inhibit oxidative stress-induced impairments [28].

While exercise and flavonoids independently show positive effects on cognitive impairment, an intriguing question is whether combining these two strategies results in synergistic effects. A longitudinal study found that combining physical activity with a diet rich in flavonoid-containing fruits and vegetables had a more significant impact on cognitive decline than either practice alone [93]. Similarly, a study on the synergistic effects of quercetin and regular treadmill exercise on Alzheimer’s disease revealed that combined exercise pretreatment and quercetin significantly improved spatial memory impairment and oxidative stress [94]. In a systematic review by Joseph et al. [95], only six out of 108 screened studies explored the combined intervention of flavonoids and exercise. However, one study demonstrated that the combined action of exercise and a polyphenol-enriched diet could effectively delay or ameliorate cognitive and motor decline associated with aging by improving monoaminergic neurotransmitters and increasing SIRT1 levels in key cognitive regions [96].

This protocol paper outlines the methodology for evaluating the combined effect of *C. caudatus* intake and treadmill exercise on aluminum chloride (AlCl_3_)-induced MCI. *C. caudatus* supplements may potentially reduce lipid peroxidation, as indicated by TBARS biomarkers, and increase serum SOD and glutathione levels. They may also improve global cognitive function in older adults with MCI [28]. This protocol outlines a study designed to determine whether *C. caudatus* supplementation combined with exercise training influences neurobehavioural outcomes, biomarkers, and brain histology in AlCl_3_-induced rats. Based on earlier findings from related studies, we hypothesize that the combined intervention may offer enhanced benefits compared to either approach alone, particularly in relation to spatial memory and oxidative stress parameters. These hypotheses are intended to guide the methodological framework of the study rather than predict specific outcomes. The findings generated upon completion of the study may provide valuable insights into the development of accessible and cost-effective strategies aimed at supporting cognitive health in ageing populations.

## Supporting information

S1 Fig Experimental design. (DOCX)

S2 Fig Animal grouping and intervention plan. (DOCX)

S3 Fig Timeline for animal study. (DOCX)

S4 Fig Treadmill exercise for rats. (DOCX)

S1 Table List of primer sequences used in Real Time-PCR. (DOCX)
