# Supplementary material for: Effect of Cosmos Caudatus supplementation and aerobic exercise on selected neurobehaviour, biochemical profile and histology in rats with mild cognitive impairment (MCI) induced by AlCl3: Study Protocol
Source: PLoS One. 2026 Jul 8;21(7):e0349933. doi: 10.1371/journal.pone.0349933 (PMC13484953; doi:10.1371/journal.pone.0349933)
Supplement: Supplementary file 1 [file pone.0349933.s001.docx]

**Appendix**

Wistar rats (n=16)/group

**Control/ No treatment**

**Aluminium Chloride (AlCl_3_), 200 mg/kg bw (8 weeks)**

IBF

Ex & CC

Ex

CC

PBS

*C. caudatus* = 100mg/kg bw (21 days)

and/or

Treadmill exercise = 4 weeks

Homogenate (n=6)

Neurobehavioural Study

PCR analysis (n=6)

Histological Study (n=4)

Plasma Analysis

- Open Field Test (OFT)
- Novel Object Recognition (NOR)
- Y-maze
- Aβ_1-42_ level
- SOD level
- iNOS
- COX-2
- Sestrin + BDNF
- TBARS
- Protein Carbonyl
- PPAR-γ
- p38MAPK
- NF-κB/p65 levels
- miRNA NF-κB
- ChAT activity
- H&E Dyes
- Nissl Dyes
- Immunohistochemistry (AMPK)
- CRH level
- SOD
- AChE
- GSH

**Figure S1.** Experimental design.
