# Supplementary material for: Effect of Cosmos Caudatus supplementation and aerobic exercise on selected neurobehaviour, biochemical profile and histology in rats with mild cognitive impairment (MCI) induced by AlCl3: Study Protocol
Source: PLoS One. 2026 Jul 8;21(7):e0349933. doi: 10.1371/journal.pone.0349933 (PMC13484953; doi:10.1371/journal.pone.0349933)
Supplement: Supplementary file 2 [file pone.0349933.s002.docx]

**Appendix**

|  | Week 1 | Week 2 | Week 3 | Week 4 | | Week 5 | Week 6 | Week 7 | Week 8 |
| --- | --- | --- | --- | --- | --- | --- | --- | --- | --- |
| Group 1 | Aluminium chloride (AlCl3: 200 mg/kg b.wt) | | | | | | | | |
|  |  | | | | | | Normal saline | | |
| Group 2 | Aluminium chloride (AlCl3: 200 mg/kg b.wt) | | | | | | | | |
|  |  | | | | | | C. caudatus extract (100 mg/kg/b.wt) oral administration daily | | |
| Group 3 | Aluminium Chloride (AlCl3: 200 mg/kg) induction | | | | | | | | |
|  |  | | | | Treadmill training (30 min once a day, five times a week) | | | | |
| Group 4 | Aluminium Chloride (AlCl3: 200 mg/kg) induction | | | | | | | | |
|  |  | | | | Treadmill training (30 min once a day, five times a week) | | | | |
|  |  | | | | | | C. caudatus extract (100 mg/kg/b.wt) oral administration daily | | |
| Group 5 | Aluminium Chloride (AlCl3: 200 mg/kg) induction | | | | | | | | |
|  |  | | | | Ibuprofen (17.5 mg/kg) | | | | |

**Figure S2:** Animal grouping and intervention plan.
