# Supplementary material for: Effect of Cosmos Caudatus supplementation and aerobic exercise on selected neurobehaviour, biochemical profile and histology in rats with mild cognitive impairment (MCI) induced by AlCl3: Study Protocol
Source: PLoS One. 2026 Jul 8;21(7):e0349933. doi: 10.1371/journal.pone.0349933 (PMC13484953; doi:10.1371/journal.pone.0349933)
Supplement: Supplementary file 3 [file pone.0349933.s003.docx]

**Appendix**

**AlCl_3_/PBS (non-treatment) group:-**

Day 0	26	28	56	57

OFT/Y-maze	Induction	OFT/Y-maze	Administration	OFT/Y-maze	Animal

/NOR	of AlCl_3_	/NOR	of distilled water	/NOR	sacrifice

**CC group:-**

Day 0	33	35	56	57

OFT/Y-maze	Induction	OFT/Y-maze	Administration	OFT/Y-maze	Animal

/NOR	of AlCl_3_	/NOR	of C.C. /NOR	sacrifice

**Ex group:-**

Day 0	26	28	56	57

OFT/Y-maze	Induction	OFT/Y-maze	Exercise	OFT/Y-maze	Animal

/NOR	of AlCl_3_	/NOR	/NOR	sacrifice

**CC + Ex group:-**

Day 0	26	28	35	56	57

OFT/Y-maze	Induction	OFT/Y-maze	Exercise	C.C	OFT/Y-maze	Animal

/NOR	of AlCl_3_	/NOR	/NOR	sacrifice

**IBF group:-**

Day 0	26	28	56	57

OFT/Y-maze	Induction	OFT/Y-maze	IBF	OFT/Y-maze	Animal

/NOR	of AlCl_3_	/NOR	/NOR	sacrifice

**Figure S3**. Timeline for animal study.
