# Supplementary material for: Effect of Cosmos Caudatus supplementation and aerobic exercise on selected neurobehaviour, biochemical profile and histology in rats with mild cognitive impairment (MCI) induced by AlCl3: Study Protocol
Source: PLoS One. 2026 Jul 8;21(7):e0349933. doi: 10.1371/journal.pone.0349933 (PMC13484953; doi:10.1371/journal.pone.0349933)
Supplement: Supplementary file 5 [file pone.0349933.s005.docx]

**Appendix**

Gene	Primer (5’	3’)	Amplicon	TM

Size (bp)

PPAR-γ	F: CTG TTC GCC AAG GTG CTC CA	102 bp	68/4

R: GCT CAT ATC TGT CTC CGT CTT CTT

NF-κB/p65	F: TTA CGG GAG ATG TGA AGA TG	94 bp	59/9

R: ATG ATG GCT AAG TGT AGG AC

p38 MAPK	F: GCT TAC CGA TGA CCA CGA TC

R: TTC ATT CAC AGC GAG GTT GC

**Table S1:** List of primer sequences used in Real Time-PCR
