# Supplementary material for: Effect of Cosmos Caudatus supplementation and aerobic exercise on selected neurobehaviour, biochemical profile and histology in rats with mild cognitive impairment (MCI) induced by AlCl3: Study Protocol
Source: PLoS One. 2026 Jul 8;21(7):e0349933. doi: 10.1371/journal.pone.0349933 (PMC13484953; doi:10.1371/journal.pone.0349933)
Supplement: Supplementary file 6 [file pone.0349933.s006.docx]

**Effect of *Cosmos caudatus* Supplementation and Aerobic Exercise on Selected Neurobehavior, Biochemical Profile and Histology in Rats with Mild Cognitive Impairment (MCI) induced by AlCl3: Study Protocol**

The authors made appreciable efforts to study the effect of *Cosmos caudatus* but the manuscript writing is very poor and not meeting the publication standards. It should be thoroughly revised for grammatical errors and formatting errors. The manuscript may be considered for acceptance following major revisions and after considering the remaining reviewers' suggestions.

Following are the recommendations

1. Manuscript has been covered in all the tenses. All the methods were in future tense and some in past and present as well.
2. Text and paragraph formatting need to be improved.
3. Only *Cosmos caudatus* is in italics but the manuscript also contains other plant names. Change to italics.
4. The representation of the degree Celsius symbol and some units is inconsistent throughout the manuscript.
5. Mention the primer sequence used in the experiment.
6. Manuscript is not containing any data. Without data representation of the behavioral, biochemical and histopathological assays the manuscript cannot be considered further.
7. The authors used Ibuprofen in the study. It need to be mentioned the purpose of using this drug.
