# Supplementary material for: Effect of Cosmos Caudatus supplementation and aerobic exercise on selected neurobehaviour, biochemical profile and histology in rats with mild cognitive impairment (MCI) induced by AlCl3: Study Protocol
Source: PLoS One. 2026 Jul 8;21(7):e0349933. doi: 10.1371/journal.pone.0349933 (PMC13484953; doi:10.1371/journal.pone.0349933)
Supplement: Supplementary file 7 [file pone.0349933.s008.docx]

PLOS One – Response to reviewers (Version 1)

| **Manuscript ID:** | | | | |
| --- | --- | --- | --- | --- |
| Effect of Cosmos caudatus Supplementation and Aerobic Exercise on Selected Neurobehavior, Biochemical Profile and Histology in Rats with Mild Cognitive Impairment (MCI) induced by AlCl3: Study Protocol | | | | |
| Reviewer | Comment | Response | | Page |
| 2 | Following are the recommendations |  | |  |
|  | 1. Manuscript has been covered in all the tenses. All the methods were in future tense and some in past and present as well. | Correction made | |  |
|  | 2. Text and paragraph formatting need to be improved. | Correction made | |  |
|  | 3. Only Cosmos caudatus is in italics but the manuscript also contains other plant names. Change to italics. | Correction made in Introduction | | **Page 3** |
|  | 4. The representation of the degree Celsius symbol and some units is inconsistent throughout the manuscript. | Correction made | |  |
|  | 5. Mention the primer sequence used in the experiment. | Correction made and table added in supporting document | |  |
|  | 6. Manuscript is not containing any data. Without data representation of the behavioral, biochemical and histopathological assays the manuscript cannot be considered further. | Manuscript submitted is a protocol paper, as such no data has been collected by far. Once data is collected and analysis, it will be made available. | |  |
|  | 7. The authors used Ibuprofen in the study. It need to be mentioned the purpose of using this drug. | Purpose of Ibuprofen in this study has been included in methodology sub heading 2.3 Ibuprofen | | **Page 7** |
|  |  |  | |  |
| 3 | MAJOR: |  | |  |
|  | • The manuscript would benefit from a thorough review of the language and writing style. The English should be revised to maintain a more formal tone throughout. Additionally, the manuscript contains a mixture of British and American English (e.g., "behavior" vs. "behaviour"), and it is recommended that the authors choose one style and remain consistent. Additionally, the use of verb tenses is inconsistent and often incorrect. It is essential to standardize the tenses for clarity and correctness. | “behaviour” has been chosen at the preferred term and all the terms have been changed to future continuous tense | |  |
|  | • The figures are helpful in illustrating the protocol, but the Methodology section needs significant improvement. It lacks sufficient detail in some areas, making it difficult to fully understand the experimental process. Please revise this section to provide more comprehensive descriptions of the procedures and protocols. | Correction made under sub heading 2.1 Experimental Design | | **Page 6** |
|  | • Several key pieces of information that should be presented in the Introduction are instead discussed in the Discussion section. This creates a structural imbalance and confusion about the logical flow of the manuscript. Both sections should be revised to ensure that the Introduction provides background information and sets the stage for the study, while the Discussion interprets the findings and places them in the context of existing literature. | Correction made in introduction and discussion section. |  | |
|  | • There is limited information provided about Cosmos caudatus, such as its characteristics, uses, and the existing literature surrounding it, aside from the mention of its phenol-rich content with just one reference. The rationale behind choosing this plant should be explained in greater detail. | Correction made in Introduction  C. caudatus, commonly known as Ulam raja in Malaysia and Kenikir in Indonesia, is a medicinal herb belonging to the Asteraceae family, which comprises 20–26 species worldwide (Yosoff et al. 2015; Cheng et al. 2017). Traditionally consumed as a salad, it has been widely recognized for its health benefits, including antihypertensive, antidiabetic, antioxidant, antiosteoporosis, antifungal, antibacterial, and antimutagenic properties (Moshawih et al. 2017; Abdul Rahman et al. 2017; Uzbek et al. 2019). Scientific studies have validated its potential for treating diseases linked to oxidative stress, such as cataracts, atherosclerosis, diabetes (Wan-Nadilah et al. 2019), Alzheimer’s (You et al. 2021), and other neurological disorders. C. caudatus demonstrates a high total antioxidant capacity, with proanthocyanidins, quercetin glycosides, and chlorogenic acids among its major bioactive compounds (Alvarez et al. 2021). Additionally, the plant contains phenolic compounds, flavonoids, flavones, and flavanones, which contribute to its ability to scavenge free radicals and prevent oxidative damage to tissues and cells. Its diverse bioactive properties underscore its potential for therapeutic applications (Senjaya et al. 2024). | **Page 3** | |
|  | • The treatment protocols for the experimental groups lack important details. For example, in Group 2, C. caudatus extract is administered for 21 days in animals aged 6-8 weeks, but the rationale behind this treatment duration is not explained. Similarly, in Group 3, the treadmill training protocol is planned for 4 weeks, but how this duration was determined is not addressed. Given that both treatment protocols involve different lengths of treatment, the authors should explain how they plan to establish clinical relevance, particularly if a correlation between the two treatments is suggested. Additionally, for Group 5, the details regarding ibuprofen administration are unclear. The authors should specify how ibuprofen will be administered (e.g., orally or through another method), the duration of the treatment, and the dose to be used. These details are crucial for understanding the experimental design and ensuring reproducibility. | Information on reason for choosing the following added under:  Introduction  - 21 days of Cosmos caudatus supplementation  3.0 Treadmill exercise protocol   - 4 weeks of treadmill exercise   2.3 Ibuprofen   - Ibuprofen group | **Page 3**  **Page 13**  **Page 7** | |
|  | • Additionally, the brain homogenization / tissue preparation procedure is described twice (or thrice?) in the Methodology with slight differences. The authors should provide a clear and consistent explanation of the chosen protocol. | The tissue preparation method has been standardised and repetition has been removed. |  | |
|  | MINOR: |  |  | |
|  | • The abstract is semi-structured, with some sections clearly labeled with titles, while others are not. | Abstract has been structured. | **Page 1-2** | |
|  | • In Introduction: |  |  | |
|  | 1. The pharmacological treatments available modestly improve cognitive function and neuropsychiatric symptoms for limited time bus is unable to cure MCI : I believe it should be “but” instead of “bus”. | Correction made. | **Page 2** | |
|  | 2. Are there any studies on C. caudatus in animals? This study investigates a combined treatment, but more information on the individual effect of C. caudatus on MCI (Mild Cognitive Impairment) is needed to strengthen the rationale for the combination study. | There are studies on C. caudatus in animals but to our knowledge none are related to MCI. While human study on MCI showing potential effect of C. caudatus is stated in the introduction. |  | |
|  | 3. The rationale for choosing the aqueous extract was not explained. | Rationale for choosing aqueous extract have been explained in Introduction  “According to a study by Ahda et al. (2023), the pharmacological activities of *C. caudatus* largely depend on the type of extract used, as different solvents employed in the extraction process can result in variations in the bioactive metabolites obtained from the plant material. Notably, an aqueous extract of *C. caudatus* at a dose of 100 mg/kg administered for 21 days demonstrated significant antioxidant effects, highlighting its potential for therapeutic applications (Abdullah et al., 2015).” | **Page 3** | |
|  | 4. "Therefore, in-vivo animal studies are needed to determine the combined effect of C. caudatus aqueous extract and exercise on improving the biochemical profile, neurobehavior, and cognitive function of AlCl3-induced rats" – AlCl3 induces what? The authors should clarify this point for better understanding. | Correction made in Introduction  This study aims to evaluate the synergistic effects of C. caudatus supplementation and aerobic exercise on neurobehavior, biochemical profiles, and brain histology in AlCl3-induced MCI in rats. | **Page 3-4** | |
|  | 5. This section provides important information but lacks references to related studies. The authors should include citations to support the claims made. Additionally, the section needs to be revised for clarity and correct use of "time," as there are significant grammatical errors. Here's a possible revision for clarity and grammar:   "Oxidative stress in the brain manifests as lipid peroxidation, indicated by elevated levels of thiobarbituric acid-reactive substances (TBARS), and protein oxidation, indicated by increased protein carbonyl levels. Amyloid-beta (Aβ), a major contributor to Alzheimer’s disease, triggers an inflammatory response in the brain, leading to the production of various pro-inflammatory mediators such as inducible nitric oxide synthase (iNOS) and cyclooxygenase-2 (COX-2). Enzymes such as choline acetyltransferase (ChAT) and superoxide dismutase (SOD) activity are assessed in brain homogenates. In neurodegenerative diseases, a significant decrease in ChAT activity and concentration is often detected, which is followed by neuronal loss." | Correction made in Introduction  Oxidative stress in the brain manifests as lipid peroxidation, indexed by elevated levels of thiobarbituric acid reactive substances (TBARS), and protein oxidation, indicated by protein carbonyl levels. Amyloid-β (Aβ), a major etiology of Alzheimer’s disease, causes an inflammatory response in the brain, leading to the production of multiple pro-inflammatory mediators such as inducible nitric oxide synthase (iNOS) and cyclooxygenase-2 (COX-2). Enzymes such as choline acetyltransferase (ChAT) and superoxide dismutase (SOD) activity are assessed in brain homogenate. In certain neurodegenerative diseases, a significant decrease in ChAT activity and concentration is detected, followed by neuronal loss. Additionally, quercetin, a bioactive compound found in *C. caudatus*, can decrease iNOS expression (Bournival et al., 2012). | **Page 4** | |
|  | 6. The Introduction does not provide enough information about the primary endpoints of the study, specifically sestrin and BDNF. The relationship between these molecules and the current study should be explained in more detail to clarify their relevance and how they contribute to the study's objectives. | Correction made in Introduction  Evidence suggests that BDNF levels and metabolism in highly plastic brain regions, such as the cortex and hippocampus, are altered during normal aging and in Alzheimer’s disease (Dadkhah et al. 2023). For example, BDNF plasma levels are significantly reduced in elderly individuals, and BDNF expression is downregulated in the hippocampus of aged rodents (Gao et al 2022; Numakawa et al. 2022). | **Page 4** | |
|  | 7. The secondary endpoints were not clearly explained. The term "neurobehavior changes" needs further clarification—specifically, what behaviors are being assessed and how these will be tested. Additionally, the distinction between "baseline values" and "2 days before supplementation" is unclear. If these terms refer to the same time point, this should be made explicit in the text to avoid confusion. | Correction made under sub heading 1.2  Neurobehaviour changes will be assessed at baseline (day 0 before induction of AlCl_3_), after induction and 2 days before supplementation of *C. caudatus* and/or exercise training and lastly at the end of intervention before sacrifice. The neurobehaviours tested are Open Field Test (OFT) which measures locomotor activity, Y-maze which measures spatial working memory and Novel Object Recognition Test (NORT) which measures learning and memory in rats. | **Page 5** | |
|  | • In Methodology: |  |  | |
|  | 1. The study employs a model using AlCl3, but the reader is not provided with information about what this model represents, what it is used for, or how it induces the desired symptoms. The rationale for using AlCl3 should be explained to clarify why this model was chosen and how it contributes to the study's objectives. | Correction made in Introduction  Aluminum (Al) is chosen since it is the third most abundant element on Earth and is widely encountered by humans through food, water, air, dust, and medicines (Alasfar et al., 2021). Additional exposure arises from cooking or storing food in aluminum utensils and foils, as well as its use in industries such as paper production, water treatment, and pharmaceuticals (Alabi and Adeoluwa. 2020). Al is a recognized neurotoxic agent implicated in Alzheimer’s disease (AD). It has been detected in both senile plaques and neurofibrillary tangle (NFT)-bearing neurons in AD patients, suggesting its involvement in the disease (Ahmed et al. 2021). Aluminum binding enhances the penetration of Aβ proteins across the blood-brain barrier and contributes to AD-like pathological changes, including tau and Aβ accumulation, neuronal apoptosis, and disruption of iron and calcium homeostasis (Wang et al., 2021; Qin et al., 2020). Consequently, Al-induced AD models are widely used to screen potential anti-AD drugs. The primary mechanisms underlying Al-induced AD are linked to oxidative stress and autophagy dysfunction. Aluminum exposure has been shown to increase reactive oxygen species (ROS) production, induce lipid peroxidation, and impair antioxidant defenses by reducing the activity of enzymes like superoxide dismutase (SOD), catalase (CAT), and glutathione peroxidase (GPx) (Rai et al., 2023; Samman et al., 2023). | **Page 4** | |
|  | 2. What will be administered to the control group is not specified. Since the control group is still an experimental group, it should be clearly included in the protocol timelines to ensure consistency and clarity in the experimental design. | Correction made under sub heading 2.0 Methodology  Group 1: Aluminium chloride (AlCl_3_: 200 mg/kg b.wt) for 8 weeks with normal saline for 21 days and remain sedentary. | **Page 6** | |
|  | 3. The timing of the behavioral tests is not clearly outlined. The authors should specify when the behavioral tests will be conducted in relation to the treatment protocol, to ensure a clear understanding of the experimental timeline and how the tests will correlate with the interventions. | Correction made under sub heading 1.2 | **Page 5** | |
|  | 4. The rationale for using whole brains instead of specific brain segments should be explained. The authors should clarify why the entire brain was selected for analysis, as this decision could have implications for the interpretation of the results, particularly if certain brain regions are more relevant to the study's objectives. It would be helpful to reference established protocols and provide citations to support the methodology used. This will ensure transparency and reproducibility of the experimental procedures. See below the sections from the same methodology: i. Their whole brains will be separated and brain tissues will be used for histopathological examination. To prepare brain homogenate, the hippocampus from the brain samples kept at −80 °C were taken and rapidly sliced into small pieces on a cold plate. Additionally, the remaining will be kept at −40 °C for further analysis. Brain samples will be homogenized in 0.1 M phosphate buffer (pH 7.4) to yield a 10% homogenate (w/v). The homogenate will be then centrifuged at 15000xg for 30 min at 40C. The resulting supernatant obtained will be used for assaying Aβ peptide, protein carbonyl and Choline Acetyltransferase (ChAT), Inducible Nitric Oxide Synthase (iNOS), Cyclooxygenase-2 (COX-2), thiobarbituric acid-reactive substances (TBARS) assay and antioxidant enzymatic activities of Superoxide dismutase (SOD). ii. Brain samples from the control and the experimental group will be obtained. For biochemical assays, the brain will be homogenized. Tissue samples will be prepared by homogenizing 1 g of tissue in 10 ml of phosphate buffer (pH 7.4) using a manual glass homogenizer. The unbroken cells and cell debris will be removed by means of centrifugation at 3000 rpm for 10 min at 4°C. The obtained supernatants will be stored at −20°C until biochemical assays. As for histology studies, the brain will be immediately immersed in 10% buffered formalin fixative for 48 h. The specimens will be processed to obtain paraffin blocks, from which 5-μm-thick sections will be prepared and processed for histopathological and immunohistochemical studies. | Correction made under sub heading 2.1 Experimental Design  Whole brain is used for histology as the brain needs to be intact to avoid structural disruption as there will be loss of brain tissues during sectioning. This will ensure a clear view of the hippocampus area in histology assessment.  While for biomarker analysis homogenate of hippocampus will be prepared where the hippocampus from the brain samples kept at −80°C will be taken and rapidly sliced into small pieces on a cold plate. Additionally, the remaining will be kept at −40°C for further analysis. Brain samples will be homogenized in 0.1 M phosphate buffer (pH 7.4) to yield a 10% homogenate (w/v). The homogenate will be then centrifuged at 15000xg for 30 min at 40C. The resulting supernatant obtained will be used for assaying Aβ peptide, protein carbonyl and Choline Acetyltransferase (ChAT), Inducible Nitric Oxide Synthase (iNOS), Cyclooxygenase-2 (COX-2), thiobarbituric acid-reactive substances (TBARS) assay and antioxidant enzymatic activities of Superoxide dismutase (SOD). | **Page 7** | |
|  | 5. The protocol should specify that the Open Field (OF) arena will be cleaned with 70% alcohol after each rat to prevent cross-contamination and ensure accurate behavioral measurements. This detail is important for maintaining the integrity of the experimental conditions and minimizing any potential confounding effects. | Correction made under sub heading 2.5.1 Open Field Test  The arena will be wiped with 70% Ethanol prior to use and before subsequent tests to remove any scent clues left by the previous rats. Ethanol must be allowed to evaporate completely prior to testing rats. This may take 5-10 min between each testing session. | **Page 8** | |
|  | 6. The reference to "surgery" in the Novel Object Recognition Task (NOR) section is unclear. The authors should specify what surgery is being performed in this study and its relevance to the experimental design | Correction made under sub heading 2.4.2 Novel Object Recognition Task | **Page 8** | |
|  | 7. The statement "The concentration of SOD in the sample was determined by comparing the OD of the samples to the standard curve" appears in both the iNOS and COX-2 sections, which raises confusion. It should be clarified whether the authors are determining the levels of iNOS and COX-2 based on the SOD measurements, as this is not typically the case. The authors should ensure that the biochemical analysis methods for iNOS and COX-2 are distinct and properly described, and that SOD is not incorrectly associated with these measurements unless explicitly part of the methodology. Clear, separate protocols for assessing each of these biomarkers should be provided. | Correction made under sub heading 2.5 Biomarker Analysis | **Page 9** | |
|  | 8. The sections on the measurement of PPAR-γ, p38MAPK, and NF-κB/p65 levels, as well as RNA extraction and real-time PCR, are only briefly mentioned in the protocol. Providing more context about the relevance of these biomarkers in the Introduction would help increase clarity and strengthen the rationale for their inclusion in the study. The authors should briefly explain how these markers relate to the study's objectives and why they are important in investigating the effects of the interventions being tested. | Correction made under Introduction and  2.5.9 Measurement of PPAR-γ, p38MAPK, and NF-κB/p65 levels – Page 11 | **Page 5**  **Page 11** | |
|  | 9. The Histology Assessment section includes another protocol for brain tissue, but the exact separation and experimental protocol for the brains are not clearly outlined. The authors should specify how many brains will be used for histological and histopathological analyses and provide details on the separation process. For instance, will different brain regions be isolated, or will the entire brain be used for histology? Clear and detailed descriptions of these protocols are necessary to ensure reproducibility and transparency in the study's methodology. | Correction made under sub heading 2.1 Experimental Design  Approximately 24 hours after the last neurobehaviour assessment, rats will be humanely euthanized. After decapitation, whole brain will be rapidly removed. From the 16 animals in each group brains will be processed as follows:   - Four brains will be sacrificed, and their whole brains will be rapidly removed for histopathological examination.   Whole brain is used for histology as the brain needs to be intact to avoid structural disruption as there will be loss of brain tissues during sectioning. This will ensure a clear view of the hippocampus area in histology assessment. | **Page 6** | |
|  | 10. Placing the "Treadmill exercise protocol" before the histological analysis in the text would provide a more logical flow, as the exercise intervention typically occurs before any tissue analysis. This would ensure a clearer understanding of the sequence of procedures in the study. | Correction made | **Page 11** | |
|  | 11. In the treadmill protocol, the roles of the sham-operated group and the Aβ1-42-injection group should be clarified. If the study does not include a sham-operated group or an Aβ1-42 injection group, this should be clearly stated in the methodology to avoid confusion. If these groups were mentioned inadvertently or as part of a previous draft, they should be removed or replaced with the actual experimental groups used in the study. It is important for the authors to accurately describe the groups included in the study and their corresponding treatments to ensure clarity and consistency in the experimental design. | Correction made under heading 3.0 Treadmill exercise protocol | **Page 13** | |
|  | 12. The statistical analysis section should specify which post hoc test will be used after performing a one-way ANOVA, as different post hoc tests are suitable depending on the specific experimental design and comparisons being made. Common post hoc tests include Tukey's, Bonferroni, or Dunnett's test. Additionally, the authors should state the threshold P value that will be considered statistically significant, typically P < 0.05, unless otherwise specified. Providing this information ensures transparency and helps readers understand how the statistical analysis will be conducted. | Correction made under heading 6.0 Statistical Analysis  Once data collection has been completed, neurobehavior changes will be analysed using mixed design ANOVA. Also, Tukey's post-hoc test was used to compare significant differences. Furthermore, one-way ANOVA was used for assessing biochemical parameters and the statistical significance was determined by Tukey's post-hoc test. P<0.05 was regarded as statistically significant, and the results were expressed as mean ± standard deviation. | **Page 15** | |
|  | • In Discussion: |  |  | |
|  | L5: the word “crucia” is probably “crucial.” | Correction made |  | |
|  |  |  |  | |
| 4 | 1. Study the essential ten ARRIVE guidelines (E10) and ensure that all criteria and met and described in your protocol (https://arriveguidelines.org/resources/author-checklists), especially measures to increase the internal validity of your study, which includes randomization, blinding, samples size calculations and inclusion/exclusion criteria. The present protocol does not address any of these proven measures which severely threatens the internal validity of the study. Also, any analysis details are mission. | Correction made under heading 2.0 Methodology  A total of 80 adult male Wistar rats (180 ± 20 g, age: six-eight weeks) will be obtained from the animal house Faculty of Medicine, Universiti Kebangsaan Malaysia (UKM). Animals will be maintained in a 12:12-h light-dark cycle (light on between 7:00 a.m. and 7:00 p.m.) at 23±2°C and food and water will be available ad libitum. The rats will be chosen randomly. At first, eight cages will be selected and then the treatment procedure that will be written on pieces of paper will be randomly placed inside each cage. Next, the rats will be assigned one by one to different cages. Finally, animals will be divided into 5 sub-groups respectively (n=16/group) as follows in Fig 1. The animals in the same group will be housed on identical shelves in one room and treated daily for eight weeks. | **Page 12** | |
|  | 2. The experimental design provided in Figure 1 is truncated and therefore lacks clarity. Clearly designate treatment and control groups as such with the number of animals. Clearly separate tests7analyses from groups. Use a flow chart protocol, scenario 2 form this resource as guidance: https://www.protocols.io/view/how-to-design-a-study-flow-chart-dm6gpjeo8gzp/v1 A flow chart template like this will make it transparent when animals need to excluded during the run of the study. It is currently not clear for instance how many animals will be processed for fresh tissue processing or whether all brains will be fixated. The study, not the flow chart, needs to include a power calculation and effect size estimates. | Correction made under sub heading 2.1 Experimental Design | **Page 6** | |
|  | 3. In the histological assessment please describe what will be assessed for each staining and how sections per animal and condition will be analyzed. Do you employ stereological counting techniques. How else do you account for over/undercounting? check the units for the optical dissector (meter?). Why is for neuronal count a specific neuronal marker used like NeuN? A pure reliance on size difference to glia will lead to more ambiguity and less reliable results. | Explained in 4.0 (VIII) | **Page 15** | |
|  | 4. The housing of the animals is unclear? How many animals are in one cage (cage size missing) and are they of the same of different conditions of group housed? | Correction made under heading 2.0 Methodology  A total of 80 male Wistar rats 2 months of age weighing between 180-200 g, will be used in this study. Rats will be allowed food and water in ad libitium. After one week of acclimation, the rats will be randomly assigned into 2 main groups (AlCl_3_ group and control/non-treatment group) and further divided into 5 sub-groups respectively (16/group) and will be treated daily for eight weeks as follows | **Page 5** | |
|  | 5. Exercise: Provide a better description or picture of the treadmill. Are animals taken out of their home environment and forced to run for 30 min? How many animals are processed in parallel? Provide a circadian time frame when the exercises are taking place. There is no Aβ1-42 injection group mentioned anywhere else in the protocol. I assume this is a copy/paste mistake. What is the procedure for animals non-compliant with the exercise protocol? | Correction made under heading 3.0 Treadmill exercise protocol | **Page 13** | |
|  | 6. The statistical analysis is simply a description of potential tests, permitting maximal analytical flexibility which is invites bias. Clearly assign which tests will be used for which analysis! | Correction made under heading 6.0 Statistical Analysis  Once data collection has been completed, neurobehavior changes will be analysed using mixed design ANOVA. Also, Tukey's post-hoc test was used to compare significant differences. Furthermore, one-way ANOVA was used for assessing biochemical parameters and the statistical significance was determined by Tukey's post-hoc test. P<0.05 was regarded as statistically significant, and the results were expressed as mean ± standard deviation. | **Page 16** | |
|  | 7. Drug solutions: Usually, an isotonic solvent is used i.e. saline or a buffered saline. Distilled water creates hypotonic solutions should have side effects like increased pain, edema and hemolysis. Adjust the solvent for better animal welfare! | Studies using cosmos caudatus have commonly used distilled water or normal saline in control group - Senjaya et al. 2024; Abdullah et al. 2015 |  | |
|  | 8. in vitro controls: All in vitro assays should contain an appropriate control that will be always processes as an internal standard. Also consider spiking samples with known amounts of drug to ensure that there is no interference. Running a standard curve with standardized solutions in inadequate quality control. Also explain whether technical replicates will be used (duplicates or triplicates). Also define the acceptable range of values prior to the study. | Different groups are applied in the study, including both negative and positive controls. The negative control group consists of animals treated with AlCl₃ (sedentary group) without any additional treatment, while the positive control is Ibuprofen, a known drug used to treat cognitive impairment. This setup ensures that any observed effects are due to the treatment provided and that there is no interference from external factors.  Moreover, for biomarker analysis, we will utilize enzyme-linked immunosorbent assay (ELISA). All assays will be conducted in duplicates to enhance reliability and reproducibility and the specificity of the ELISA assay is ensured through the use of validated antibodies that prevent cross-reactivity, which is crucial for the accurate detection of target antigens in diverse sample types, including cells and tissue extracts. |  | |
|  | 9. tissue controls: Biochemical profiling assays should always include tissue from a control region (other than hippocampus) and/or non-brain tissue (muscle/liver, heart). Only then conclusions can be drawn regarding hippocampal and brain specificity of the results. | Previous studies have been done on other organs such as lungs, kidneys and stomach – Abdullah et al. 2015  Another study published in PLOS One Protocol entitled “Carbon monoxide as a cellular protective agent in a swine model of cardiac arrest protocol” did not include tissue from other control regions when studying on brain tissue – Greenwood et al. 2024 |  | |
|  | 10. Data availability: The manuscripts does not contain any information how , where and which data will be made openly available. In the form the authors speak of "research data" - which can be mistaken for the summarized data in a manuscript. This all contradicts the PLOS Data Policy and the best practices for transparency and reproducibility. Provide detailed information which repository will be used to deposit raw and processed data, under what license and what you are going to do produce data compliant with the FAIR standards. | Correction made under heading 6.0 Statistical Analysis | **Page 16** | |
|  | 11. Provide all data from the chromatographic analysis of C. caudatus extract that is given via oral gavage. Only then inferences can be made about active ingredients that can be extracted and tested in a subsequent study. | Correction made under sub heading 2.4 Cosmos caudatus preparation and supplementation  Liquid chromatography–mass spectrometry (LC–MS) will be done to identify the bioactive compounds in C. caudatus prior to inducing the supplement to the rats to ensure the outcome is due to the active ingredients found in C. caudatus. | **Page 8** | |
|  |  |  |  | |
| 5 | kindly revise the method part of (2.5.9) and mention the manufacturer's protocol. Also, the abstract is different from the introduction and based on pubmed search that were not mentioned in the introduction part. Rewrite the primary endpoints as sentences instead of bullet points. | Correction made under sub heading 2.5.9 Measurement of PPAR-γ, p38MAPK, and NF-κB/p65 levels | **Page 11** | |
