# Supplementary material for: Effect of Cosmos Caudatus supplementation and aerobic exercise on selected neurobehaviour, biochemical profile and histology in rats with mild cognitive impairment (MCI) induced by AlCl3: Study Protocol
Source: PLoS One. 2026 Jul 8;21(7):e0349933. doi: 10.1371/journal.pone.0349933 (PMC13484953; doi:10.1371/journal.pone.0349933)
Supplement: Supplementary file 8 [file pone.0349933.s009.docx]

Response to reviewers

| No | Issues Raised | Response |
| --- | --- | --- |
| 1 | Please include a figure label and title for Figure 3 in your main manuscript. | We respectfully wish to clarify that the requested revision on including figure label and title for Figure 3 has already been fully addressed in our revised manuscript.  To assist further, we have highlighted the relevant revision again (in yellow) and refer you to page 8 of the manuscript for ease of reference. |
| 2 | Please upload a copy of Figure 3 which you refer to in your text on page 13. Or if the figure is no longer to be included as part of the submission please remove all reference to it within the text. | Specifically, we made the changes as per the reviewer’s suggestion in our previous submission. The corresponding section(s) have been clearly updated in the manuscript and were also detailed in our response letter to the reviewers.  To assist further, we have highlighted the relevant revision again (in yellow) of the manuscript for ease of reference. We hope this resolves any misunderstanding and thank you for your attention to this clarification. |
