# Supplementary material for: Effect of Cosmos Caudatus supplementation and aerobic exercise on selected neurobehaviour, biochemical profile and histology in rats with mild cognitive impairment (MCI) induced by AlCl3: Study Protocol
Source: PLoS One. 2026 Jul 8;21(7):e0349933. doi: 10.1371/journal.pone.0349933 (PMC13484953; doi:10.1371/journal.pone.0349933)
Supplement: Peer review history

# Peer review: Decision Letter 0

**DOI:** 10.1371/journal.pone.0349933.r001

**Authors:** Vara Prasad Saka

26 Nov 2024

PONE-D-24-27017

Effect of Cosmos caudatus Supplementation and Aerobic Exercise on Selected Neurobehavior, Biochemical Profile and Histology in Rats with Mild Cognitive Impairment (MCI) induced by AlCl3: Study Protocol

PLOS ONE

Dear Dr. Joseph,

Thank you for submitting your manuscript to PLOS ONE. After careful consideration, we feel that it has merit but does not fully meet PLOS ONE’s publication criteria as it currently stands. Therefore, we invite you to submit a revised version of the manuscript that addresses the points raised during the review process.

Please submit your revised manuscript by Jan 10 2025 11:59PM. If you will need more time than this to complete your revisions, please reply to this message or contact the journal office at plosone@plos.org. When you're ready to submit your revision, log on to https://www.editorialmanager.com/pone/ and select the 'Submissions Needing Revision' folder to locate your manuscript file.

Please include the following items when submitting your revised manuscript:

- A rebuttal letter that responds to each point raised by the academic editor and reviewer(s). You should upload this letter as a separate file labeled 'Response to Reviewers'.
- A marked-up copy of your manuscript that highlights changes made to the original version. You should upload this as a separate file labeled 'Revised Manuscript with Track Changes'.
- An unmarked version of your revised paper without tracked changes. You should upload this as a separate file labeled 'Manuscript'.

If you would like to make changes to your financial disclosure, please include your updated statement in your cover letter. Guidelines for resubmitting your figure files are available below the reviewer comments at the end of this letter.

If applicable, we recommend that you deposit your laboratory protocols in protocols.io to enhance the reproducibility of your results. Protocols.io assigns your protocol its own identifier (DOI) so that it can be cited independently in the future. For instructions see: https://journals.plos.org/plosone/s/submission-guidelines#loc-laboratory-protocols. Additionally, PLOS ONE offers an option for publishing peer-reviewed Lab Protocol articles, which describe protocols hosted on protocols.io. Read more information on sharing protocols at https://plos.org/protocols?utm_medium=editorial-email&utm_source=authorletters&utm_campaign=protocols.

We look forward to receiving your revised manuscript.

Kind regards,

Vara Prasad Saka

Academic Editor

PLOS ONE

Journal Requirements:

When submitting your revision, we need you to address these additional requirements.

1. Please ensure that your manuscript meets PLOS ONE's style requirements, including those for file naming. The PLOS ONE style templates can be found at

https://journals.plos.org/plosone/s/file?id=wjVg/PLOSOne_formatting_sample_main_body.pdf and

https://journals.plos.org/plosone/s/file?id=ba62/PLOSOne_formatting_sample_title_authors_affiliations.pdf

2. Please provide a complete Data Availability Statement in the submission form, ensuring you include all necessary access information or a reason for why you are unable to make your data freely accessible. If your research concerns only data provided within your submission, please write "All data are in the manuscript and/or supporting information files" as your Data Availability Statement.

3. Please include captions for your Supporting Information files at the end of your manuscript, and update any in-text citations to match accordingly. Please see our Supporting Information guidelines for more information: http://journals.plos.org/plosone/s/supporting-information.

[Note: HTML markup is below. Please do not edit.]

Reviewers' comments:

Reviewer's Responses to Questions

**Comments to the Author**

1. Does the manuscript provide a valid rationale for the proposed study, with clearly identified and justified research questions?

The research question outlined is expected to address a valid academic problem or topic and contribute to the base of knowledge in the field.

Reviewer #1: Yes

Reviewer #2: Partly

Reviewer #3: Partly

Reviewer #4: Yes

Reviewer #5: Yes

**********

2. Is the protocol technically sound and planned in a manner that will lead to a meaningful outcome and allow testing the stated hypotheses?

The manuscript should describe the methods in sufficient detail to prevent undisclosed flexibility in the experimental procedure or analysis pipeline, including sufficient outcome-neutral conditions (e.g. necessary controls, absence of floor or ceiling effects) to test the proposed hypotheses and a statistical power analysis where applicable. As there may be aspects of the methodology and analysis which can only be refined once the work is undertaken, authors should outline potential assumptions and explicitly describe what aspects of the proposed analyses, if any, are exploratory.

Reviewer #1: Partly

Reviewer #2: Yes

Reviewer #3: Partly

Reviewer #4: Partly

Reviewer #5: Yes

**********

3. Is the methodology feasible and described in sufficient detail to allow the work to be replicable?

Descriptions of methods and materials in the protocol should be reported in sufficient detail for another researcher to reproduce all experiments and analyses. The protocol should describe the appropriate controls, sample size calculations, and replication needed to ensure that the data are robust and reproducible.

Reviewer #1: Yes

Reviewer #2: Yes

Reviewer #3: No

Reviewer #4: No

Reviewer #5: Yes

**********

4. Have the authors described where all data underlying the findings will be made available when the study is complete?

The PLOS Data policy requires authors to make all data underlying the findings described in their manuscript fully available without restriction, with rare exception, at the time of publication. The data should be provided as part of the manuscript or its supporting information, or deposited to a public repository. For example, in addition to summary statistics, the data points behind means, medians and variance measures should be available. If there are restrictions on publicly sharing data—e.g. participant privacy or use of data from a third party—those must be specified.

Reviewer #1: Yes

Reviewer #2: No

Reviewer #3: Yes

Reviewer #4: No

Reviewer #5: No

**********

5. Is the manuscript presented in an intelligible fashion and written in standard English?

PLOS ONE does not copyedit accepted manuscripts, so the language in submitted articles must be clear, correct, and unambiguous. Any typographical or grammatical errors should be corrected at revision, so please note any specific errors here.

Reviewer #1: Yes

Reviewer #2: No

Reviewer #3: No

Reviewer #4: Yes

Reviewer #5: Yes

**********

6. Review Comments to the Author

Please use the space provided to explain your answers to the questions above and, if applicable, provide comments about issues authors must address before this protocol can be accepted for publication. You may also include additional comments for the author, including concerns about research or publication ethics.

You may also provide optional suggestions and comments to authors that they might find helpful in planning their study.

(Please upload your review as an attachment if it exceeds 20,000 characters)

Reviewer #1: The manuscript is well conceived and merits publication subject to

technical changes in the introduction and discussion sections. Authors

are requested to kindly look up the formatting aspects of references once again

prior to resubmission. Best wishes.

Reviewer #2: Effect of Cosmos caudatus Supplementation and Aerobic Exercise on Selected Neurobehavior, Biochemical Profile and Histology in Rats with Mild Cognitive Impairment (MCI) induced by AlCl3: Study Protocol

The authors made appreciable efforts to study the effect of Cosmos caudatus but the manuscript writing is very poor and not meeting the publication standards. It should be thoroughly revised for grammatical errors and formatting errors. The manuscript may be considered for acceptance following major revisions and after considering the remaining reviewers' suggestions.

Following are the recommendations

1. Manuscript has been covered in all the tenses. All the methods were in future tense and some in past and present as well.

2. Text and paragraph formatting need to be improved.

3. Only Cosmos caudatus is in italics but the manuscript also contains other plant names. Change to italics.

4. The representation of the degree Celsius symbol and some units is inconsistent throughout the manuscript.

5. Mention the primer sequence used in the experiment.

6. Manuscript is not containing any data. Without data representation of the behavioral, biochemical and histopathological assays the manuscript cannot be considered further.

7. The authors used Ibuprofen in the study. It need to be mentioned the purpose of using this drug.

Reviewer #3: The authors presented a study protocol that is both comprehensive and valuable. I believe the manuscript has potential but would benefit from significant revisions. There are several areas where clarity, depth, and attention to detail could be improved. The authors may want to reconsider some sections to address missing key points that would strengthen the manuscript. My suggestions are intended to guide improvements and help make the manuscript more robust and impactful.

Please find below my comments:

MAJOR:

• The manuscript would benefit from a thorough review of the language and writing style. The English should be revised to maintain a more formal tone throughout. Additionally, the manuscript contains a mixture of British and American English (e.g., "behavior" vs. "behaviour"), and it is recommended that the authors choose one style and remain consistent. Additionally, the use of verb tenses is inconsistent and often incorrect. It is essential to standardize the tenses for clarity and correctness.

• The figures are helpful in illustrating the protocol, but the Methodology section needs significant improvement. It lacks sufficient detail in some areas, making it difficult to fully understand the experimental process. Please revise this section to provide more comprehensive descriptions of the procedures and protocols.

• Several key pieces of information that should be presented in the Introduction are instead discussed in the Discussion section. This creates a structural imbalance and confusion about the logical flow of the manuscript. Both sections should be revised to ensure that the Introduction provides background information and sets the stage for the study, while the Discussion interprets the findings and places them in the context of existing literature.

• There is limited information provided about Cosmos caudatus, such as its characteristics, uses, and the existing literature surrounding it, aside from the mention of its phenol-rich content with just one reference. The rationale behind choosing this plant should be explained in greater detail.

• The treatment protocols for the experimental groups lack important details. For example, in Group 2, C. caudatus extract is administered for 21 days in animals aged 6-8 weeks, but the rationale behind this treatment duration is not explained. Similarly, in Group 3, the treadmill training protocol is planned for 4 weeks, but how this duration was determined is not addressed. Given that both treatment protocols involve different lengths of treatment, the authors should explain how they plan to establish clinical relevance, particularly if a correlation between the two treatments is suggested. Additionally, for Group 5, the details regarding ibuprofen administration are unclear. The authors should specify how ibuprofen will be administered (e.g., orally or through another method), the duration of the treatment, and the dose to be used. These details are crucial for understanding the experimental design and ensuring reproducibility.

• Additionally, the brain homogenization / tissue preparation procedure is described twice (or thrice?) in the Methodology with slight differences. The authors should provide a clear and consistent explanation of the chosen protocol.

MINOR:

• The abstract is semi-structured, with some sections clearly labeled with titles, while others are not.

• In Introduction:

 The pharmacological treatments available modestly improve cognitive function and neuropsychiatric symptoms for limited time bus is unable to cure MCI : I believe it should be “but” instead of “bus”.

 Are there any studies on C. caudatus in animals? This study investigates a combined treatment, but more information on the individual effect of C. caudatus on MCI (Mild Cognitive Impairment) is needed to strengthen the rationale for the combination study.

 The rationale for choosing the aqueous extract was not explained.

 "Therefore, in-vivo animal studies are needed to determine the combined effect of C. caudatus aqueous extract and exercise on improving the biochemical profile, neurobehavior, and cognitive function of AlCl3-induced rats" – AlCl3 induces what? The authors should clarify this point for better understanding.

 This section provides important information but lacks references to related studies. The authors should include citations to support the claims made. Additionally, the section needs to be revised for clarity and correct use of "time," as there are significant grammatical errors. Here's a possible revision for clarity and grammar:

"Oxidative stress in the brain manifests as lipid peroxidation, indicated by elevated levels of thiobarbituric acid-reactive substances (TBARS), and protein oxidation, indicated by increased protein carbonyl levels. Amyloid-beta (Aβ), a major contributor to Alzheimer’s disease, triggers an inflammatory response in the brain, leading to the production of various pro-inflammatory mediators such as inducible nitric oxide synthase (iNOS) and cyclooxygenase-2 (COX-2). Enzymes such as choline acetyltransferase (ChAT) and superoxide dismutase (SOD) activity are assessed in brain homogenates. In neurodegenerative diseases, a significant decrease in ChAT activity and concentration is often detected, which is followed by neuronal loss."

 The Introduction does not provide enough information about the primary endpoints of the study, specifically sestrin and BDNF. The relationship between these molecules and the current study should be explained in more detail to clarify their relevance and how they contribute to the study's objectives.

 The secondary endpoints were not clearly explained. The term "neurobehavior changes" needs further clarification—specifically, what behaviors are being assessed and how these will be tested. Additionally, the distinction between "baseline values" and "2 days before supplementation" is unclear. If these terms refer to the same time point, this should be made explicit in the text to avoid confusion.

• In Methodology:

 The study employs a model using AlCl3, but the reader is not provided with information about what this model represents, what it is used for, or how it induces the desired symptoms. The rationale for using AlCl3 should be explained to clarify why this model was chosen and how it contributes to the study's objectives.

 What will be administered to the control group is not specified. Since the control group is still an experimental group, it should be clearly included in the protocol timelines to ensure consistency and clarity in the experimental design.

 The timing of the behavioral tests is not clearly outlined. The authors should specify when the behavioral tests will be conducted in relation to the treatment protocol, to ensure a clear understanding of the experimental timeline and how the tests will correlate with the interventions.

 The rationale for using whole brains instead of specific brain segments should be explained. The authors should clarify why the entire brain was selected for analysis, as this decision could have implications for the interpretation of the results, particularly if certain brain regions are more relevant to the study's objectives. It would be helpful to reference established protocols and provide citations to support the methodology used. This will ensure transparency and reproducibility of the experimental procedures. See below the sections from the same methodology:

1. Their whole brains will be separated and brain tissues will be used for histopathological examination. To prepare brain homogenate, the hippocampus from the brain samples kept at −80 °C were taken and rapidly sliced into small pieces on a cold plate. Additionally, the remaining will be kept at −40 °C for further analysis. Brain samples will be homogenized in 0.1 M phosphate buffer (pH 7.4) to yield a 10% homogenate (w/v). The homogenate will be then centrifuged at 15000xg for 30 min at 40C. The resulting supernatant obtained will be used for assaying Aβ peptide, protein carbonyl and Choline Acetyltransferase (ChAT), Inducible Nitric Oxide Synthase (iNOS), Cyclooxygenase-2 (COX-2), thiobarbituric acid-reactive substances (TBARS) assay and antioxidant enzymatic activities of Superoxide dismutase (SOD).

2. Brain samples from the control and the experimental group will be obtained. For biochemical assays, the brain will be homogenized. Tissue samples will be prepared by homogenizing 1 g of tissue in 10 ml of phosphate buffer (pH 7.4) using a manual glass homogenizer. The unbroken cells and cell debris will be removed by means of centrifugation at 3000 rpm for 10 min at 4°C. The obtained supernatants will be stored at −20°C until biochemical assays. As for histology studies, the brain will be immediately immersed in 10% buffered formalin fixative for 48 h. The specimens will be processed to obtain paraffin blocks, from which 5-μm-thick sections will be prepared and processed for histopathological and immunohistochemical studies.

 The number of researchers who will observe the recordings and score the behaviors should be specified. The authors should clarify whether one or multiple researchers will be involved in scoring, and how inter-rater reliability will be ensured to maintain consistency and accuracy in the behavioral assessments. This is important to reduce bias and increase the credibility of the results.

 The protocol should specify that the Open Field (OF) arena will be cleaned with 70% alcohol after each rat to prevent cross-contamination and ensure accurate behavioral measurements. This detail is important for maintaining the integrity of the experimental conditions and minimizing any potential confounding effects.

 The reference to "surgery" in the Novel Object Recognition Task (NOR) section is unclear. The authors should specify what surgery is being performed in this study and its relevance to the experimental design.

 The statement "The concentration of SOD in the sample was determined by comparing the OD of the samples to the standard curve" appears in both the iNOS and COX-2 sections, which raises confusion. It should be clarified whether the authors are determining the levels of iNOS and COX-2 based on the SOD measurements, as this is not typically the case. The authors should ensure that the biochemical analysis methods for iNOS and COX-2 are distinct and properly described, and that SOD is not incorrectly associated with these measurements unless explicitly part of the methodology. Clear, separate protocols for assessing each of these biomarkers should be provided.

 The sections on the measurement of PPAR-γ, p38MAPK, and NF-κB/p65 levels, as well as RNA extraction and real-time PCR, are only briefly mentioned in the protocol. Providing more context about the relevance of these biomarkers in the Introduction would help increase clarity and strengthen the rationale for their inclusion in the study. The authors should briefly explain how these markers relate to the study's objectives and why they are important in investigating the effects of the interventions being tested.

 The Histology Assessment section includes another protocol for brain tissue, but the exact separation and experimental protocol for the brains are not clearly outlined. The authors should specify how many brains will be used for histological and histopathological analyses and provide details on the separation process. For instance, will different brain regions be isolated, or will the entire brain be used for histology? Clear and detailed descriptions of these protocols are necessary to ensure reproducibility and transparency in the study's methodology.

 Placing the "Treadmill exercise protocol" before the histological analysis in the text would provide a more logical flow, as the exercise intervention typically occurs before any tissue analysis. This would ensure a clearer understanding of the sequence of procedures in the study.

 In the treadmill protocol, the roles of the sham-operated group and the Aβ1-42-injection group should be clarified. If the study does not include a sham-operated group or an Aβ1-42 injection group, this should be clearly stated in the methodology to avoid confusion. If these groups were mentioned inadvertently or as part of a previous draft, they should be removed or replaced with the actual experimental groups used in the study. It is important for the authors to accurately describe the groups included in the study and their corresponding treatments to ensure clarity and consistency in the experimental design.

 The statistical analysis section should specify which post hoc test will be used after performing a one-way ANOVA, as different post hoc tests are suitable depending on the specific experimental design and comparisons being made. Common post hoc tests include Tukey's, Bonferroni, or Dunnett's test. Additionally, the authors should state the threshold P value that will be considered statistically significant, typically P < 0.05, unless otherwise specified. Providing this information ensures transparency and helps readers understand how the statistical analysis will be conducted.

• In Discussion:

 L5: the word “crucia” is probably “crucial.”

Reviewer #4: I reviewed the study protocol for a planned preclinical study by Joseph et al. with the title: Effect of Cosmos caudatus and Aerobic Exercise in rats with Mild Cognitive Impairment(MCI) induced by AlCl3. The rationale and study are well described to understand the undertaking and execute the study. However, the quality criteria of the present study do not correspond with current criteria and recommended reporting guidelines. Therefore, I recommend sincere changes to adhere to common and current standards to produce a study of value.

1. Study the essential ten ARRIVE guidelines (E10) and ensure that all criteria and met and described in your protocol (https://arriveguidelines.org/resources/author-checklists), especially measures to increase the internal validity of your study, which includes randomization, blinding, samples size calculations and inclusion/exclusion criteria. The present protocol does not address any of these proven measures which severely threatens the internal validity of the study. Also, any analysis details are mission.

2. The experimental design provided in Figure 1 is truncated and therefore lacks clarity. Clearly designate treatment and control groups as such with the number of animals. Clearly separate tests7analyses from groups. Use a flow chart protocol, scenario 2 form this resource as guidance: https://www.protocols.io/view/how-to-design-a-study-flow-chart-dm6gpjeo8gzp/v1 A flow chart template like this will make it transparent when animals need to excluded during the run of the study. It is currently not clear for instance how many animals will be processed for fresh tissue processing or whether all brains will be fixated. The study, not the flow chart, needs to include a power calculation and effect size estimates.

3. In the histological assessment please describe what will be assessed for each staining and how sections per animal and condition will be analyzed. Do you employ stereological counting techniques. How else do you account for over/undercounting? check the units for the optical dissector (meter?). Why is for neuronal count a specific neuronal marker used like NeuN? A pure reliance on size difference to glia will lead to more ambiguity and less reliable results.

4. The housing of the animals is unclear? How many animals are in one cage (cage size missing) and are they of the same of different conditions of group housed?

5. Exercise: Provide a better description or picture of the treadmill. Are animals taken out of their home environment and forced to run for 30 min? How many animals are processed in parallel? Provide a circadian time frame when the exercises are taking place. There is no Aβ1-42 injection group mentioned anywhere else in the protocol. I assume this is a copy/paste mistake. What is the procedure for animals non-compliant with the exercise protocol?

6. The statistical analysis is simply a description of potential tests, permitting maximal analytical flexibility which is invites bias. Clearly assign which tests will be used for which analysis!

7. Drug solutions: Usually, an isotonic solvent is used i.e. saline or a buffered saline. Distilled water creates hypotonic solutions should have side effects like increased pain, edema and hemolysis. Adjust the solvent for better animal welfare!

8. in vitro controls: All in vitro assays should contain an appropriate control that will be always processes as an internal standard. Also consider spiking samples with known amounts of drug to ensure that there is no interference. Running a standard curve with standardized solutions in inadequate quality control. Also explain whether technical replicates will be used (duplicates or triplicates). Also define the acceptable range of values prior to the study.

9. tissue controls: Biochemical profiling assays should always include tissue from a control region (other than hippocampus) and/or non-brain tissue (muscle/liver, heart). Only then conclusions can be drawn regarding hippocampal and brain specificity of the results.

10. Data availability: The manuscripts does not contain any information how , where and which data will be made openly available. In the form the authors speak of "research data" - which can be mistaken for the summarized data in a manuscript. This all contradicts the PLOS Data Policy and the best practices for transparency and reproducibility. Provide detailed information which repository will be used to deposit raw and processed data, under what license and what you are going to do produce data compliant with the FAIR standards.

11. Provide all data from the chromatographic analysis of C. caudatus extract that is given via oral gavage. Only then inferences can be made about active ingredients that can be extracted and tested in a subsequent study.

Reviewer #5: Great work! The paper's protocol gave a new insight to evaluate MCI induced by AlCl3, however, kindly revise the method part of (2.5.9) and mention the manufacturer's protocol. Also, the abstract is different from the introduction and based on pubmed search that were not mentioned in the introduction part. Rewrite the primary endpoints as sentences instead of bullet points.

**********

7. PLOS authors have the option to publish the peer review history of their article (what does this mean?). If published, this will include your full peer review and any attached files.

If you choose “no”, your identity will remain anonymous but your review may still be made public.

**Do you want your identity to be public for this peer review?**  For information about this choice, including consent withdrawal, please see our Privacy Policy.

Reviewer #1: **Yes:**  Raja Chakraverty

Reviewer #2: **Yes:**  Geetanjali Devabattula

Reviewer #3: **Yes:**  Yagmur Okcay

Reviewer #4: **Yes:**  René Bernard

Reviewer #5: No

**********

[NOTE: If reviewer comments were submitted as an attachment file, they will be attached to this email and accessible via the submission site. Please log into your account, locate the manuscript record, and check for the action link "View Attachments". If this link does not appear, there are no attachment files.]

While revising your submission, please upload your figure files to the Preflight Analysis and Conversion Engine (PACE) digital diagnostic tool, https://pacev2.apexcovantage.com/. PACE helps ensure that figures meet PLOS requirements. To use PACE, you must first register as a user. Registration is free. Then, login and navigate to the UPLOAD tab, where you will find detailed instructions on how to use the tool. If you encounter any issues or have any questions when using PACE, please email PLOS at figures@plos.org. Please note that Supporting Information files do not need this step.

Attachment Submitted filename: Comments.docx

# Author comment: Author response to Decision Letter 1

**DOI:** 10.1371/journal.pone.0349933.r002

30 Apr 2025

A document detailing the response to the reviewers have been attached.

Attachment Submitted filename: Response To reviewer Round 250125.docx

# Peer review: Decision Letter 1

**DOI:** 10.1371/journal.pone.0349933.r003

**Authors:** Vara Prasad Saka, John Falode

19 Aug 2025

PONE-D-24-27017R1

Effect of Cosmos caudatus Supplementation and Aerobic Exercise on Selected Neurobehavior, Biochemical Profile and Histology in Rats with Mild Cognitive Impairment (MCI) induced by AlCl3: Study Protocol

PLOS ONE

Dear Dr. Joseph,

Thank you for submitting your manuscript to PLOS ONE. After careful consideration, we feel that it has merit but does not fully meet PLOS ONE’s publication criteria as it currently stands. Therefore, we invite you to submit a revised version of the manuscript that addresses the points raised during the review process.

==============================

1. Under experimental design, from groups 2 to 5, are the treatment options administered together with the AlCl3 induction for 8 weeks? Or are they administered after the AlCl3 induction? So, the authors need to state clearly if it is pre-treatment, co-administration, or post-treatment.

These are very important points to define.

==============================

Please submit your revised manuscript by Oct 03 2025 11:59PM. If you will need more time than this to complete your revisions, please reply to this message or contact the journal office at plosone@plos.org. When you're ready to submit your revision, log on to https://www.editorialmanager.com/pone/ and select the 'Submissions Needing Revision' folder to locate your manuscript file.

Please include the following items when submitting your revised manuscript:

- A rebuttal letter that responds to each point raised by the academic editor and reviewer(s). You should upload this letter as a separate file labeled 'Response to Reviewers'.
- A marked-up copy of your manuscript that highlights changes made to the original version. You should upload this as a separate file labeled 'Revised Manuscript with Track Changes'.
- An unmarked version of your revised paper without tracked changes. You should upload this as a separate file labeled 'Manuscript'.

If you would like to make changes to your financial disclosure, please include your updated statement in your cover letter. Guidelines for resubmitting your figure files are available below the reviewer comments at the end of this letter.

If applicable, we recommend that you deposit your laboratory protocols in protocols.io to enhance the reproducibility of your results. Protocols.io assigns your protocol its own identifier (DOI) so that it can be cited independently in the future. For instructions see: https://journals.plos.org/plosone/s/submission-guidelines#loc-laboratory-protocols. Additionally, PLOS ONE offers an option for publishing peer-reviewed Lab Protocol articles, which describe protocols hosted on protocols.io. Read more information on sharing protocols at https://plos.org/protocols?utm_medium=editorial-email&utm_source=authorletters&utm_campaign=protocols.

We look forward to receiving your revised manuscript.

Kind regards,

John Adeolu Falode

Academic Editor

PLOS ONE

Journal Requirements:

If the reviewer comments include a recommendation to cite specific previously published works, please review and evaluate these publications to determine whether they are relevant and should be cited. There is no requirement to cite these works unless the editor has indicated otherwise.

[Note: HTML markup is below. Please do not edit.]

Reviewers' comments:

Reviewer's Responses to Questions

**Comments to the Author**

1. Does the manuscript provide a valid rationale for the proposed study, with clearly identified and justified research questions?

The research question outlined is expected to address a valid academic problem or topic and contribute to the base of knowledge in the field.

Reviewer #1: No

Reviewer #3: No

Reviewer #4: Yes

**********

2. Is the protocol technically sound and planned in a manner that will lead to a meaningful outcome and allow testing the stated hypotheses?

The manuscript should describe the methods in sufficient detail to prevent undisclosed flexibility in the experimental procedure or analysis pipeline, including sufficient outcome-neutral conditions (e.g. necessary controls, absence of floor or ceiling effects) to test the proposed hypotheses and a statistical power analysis where applicable. As there may be aspects of the methodology and analysis which can only be refined once the work is undertaken, authors should outline potential assumptions and explicitly describe what aspects of the proposed analyses, if any, are exploratory.

Reviewer #1: No

Reviewer #3: Partly

Reviewer #4: Yes

**********

3. Is the methodology feasible and described in sufficient detail to allow the work to be replicable?

Descriptions of methods and materials in the protocol should be reported in sufficient detail for another researcher to reproduce all experiments and analyses. The protocol should describe the appropriate controls, sample size calculations, and replication needed to ensure that the data are robust and reproducible.

Reviewer #1: No

Reviewer #3: No

Reviewer #4: Yes

**********

4. Have the authors described where all data underlying the findings will be made available when the study is complete?

The PLOS Data policy requires authors to make all data underlying the findings described in their manuscript fully available without restriction, with rare exception, at the time of publication. The data should be provided as part of the manuscript or its supporting information, or deposited to a public repository. For example, in addition to summary statistics, the data points behind means, medians and variance measures should be available. If there are restrictions on publicly sharing data—e.g. participant privacy or use of data from a third party—those must be specified.

Reviewer #1: No

Reviewer #3: Yes

Reviewer #4: Yes

**********

5. Is the manuscript presented in an intelligible fashion and written in standard English?

PLOS ONE does not copyedit accepted manuscripts, so the language in submitted articles must be clear, correct, and unambiguous. Any typographical or grammatical errors should be corrected at revision, so please note any specific errors here.

Reviewer #1: Yes

Reviewer #3: Yes

Reviewer #4: Yes

**********

6. Review Comments to the Author

Please use the space provided to explain your answers to the questions above and, if applicable, provide comments about issues authors must address before this protocol can be accepted for publication. You may also include additional comments for the author, including concerns about research or publication ethics.

You may also provide optional suggestions and comments to authors that they might find helpful in planning their study.

(Please upload your review as an attachment if it exceeds 20,000 characters)

Reviewer #1: Serious similarity indices suspected and suggestions have not been adequately

addressed and have serious drawbacks in methodology and hence the decision was prompted.

Reviewer #3: I would like to begin by thanking the authors for making the necessary revisions to the manuscript. The changes have clearly improved the scientific clarity and structure of the study, and I appreciate the effort invested in refining the work.

However, after careful evaluation, I believe that this manuscript is more appropriate as a research article rather than a study protocol. While the study is well-conceived and addresses an important topic, a protocol article is expected to present a clear, reproducible, and generally applicable methodological framework—prior to data collection—and without the inclusion or implication of results. In this manuscript, the authors detail numerous procedures, including behavioral assessments and ELISA analyses, yet no results are provided. Even if results were included, they would pertain specifically to Cosmos caudatus and would not offer generalizable insights applicable across different interventions.

Furthermore, a study protocol should emphasize standardized procedures that can be replicated or adapted in broader research contexts. The current manuscript focuses on a single plant extract with study-specific methodology, which limits its utility as a widely applicable protocol. The lack of methodological generalizability, combined with the inclusion of detailed experimental steps designed for a specific compound, ultimately makes this more suitable as a research article.

Therefore, despite the merits of the study itself, I regret to recommend rejection in its current form as a study protocol. I encourage the authors to consider revising and submitting it as a full research article, where the strength of their experimental design and findings can be properly showcased.

Reviewer #4: I thank the authors for submitting a revised protocol which is much improved now and I recommend the acceptance of your protocol for publication.

**********

7. PLOS authors have the option to publish the peer review history of their article (what does this mean?). If published, this will include your full peer review and any attached files.

If you choose “no”, your identity will remain anonymous but your review may still be made public.

**Do you want your identity to be public for this peer review?**  For information about this choice, including consent withdrawal, please see our Privacy Policy.

Reviewer #1: **Yes:**  Raja Chakraverty

Reviewer #3: **Yes:**  Yagmur Okcay

Reviewer #4: **Yes:**  René Bernard

**********

[NOTE: If reviewer comments were submitted as an attachment file, they will be attached to this email and accessible via the submission site. Please log into your account, locate the manuscript record, and check for the action link "View Attachments". If this link does not appear, there are no attachment files.]

While revising your submission, please upload your figure files to the Preflight Analysis and Conversion Engine (PACE) digital diagnostic tool, https://pacev2.apexcovantage.com/. PACE helps ensure that figures meet PLOS requirements. To use PACE, you must first register as a user. Registration is free. Then, login and navigate to the UPLOAD tab, where you will find detailed instructions on how to use the tool. If you encounter any issues or have any questions when using PACE, please email PLOS at figures@plos.org. Please note that Supporting Information files do not need this step.

# Author comment: Author response to Decision Letter 2

**DOI:** 10.1371/journal.pone.0349933.r004

25 Nov 2025

Response to reviewers have been attached as a separate document.

Attachment Submitted filename: Response to reviewers Round 4.docx

# Peer review: Decision Letter 2

**DOI:** 10.1371/journal.pone.0349933.r005

**Authors:** Vara Prasad Saka, John Falode, Hira Rafi

10 Feb 2026

PONE-D-24-27017R2

Effect of Cosmos caudatus Supplementation and Aerobic Exercise on Selected Neurobehavior, Biochemical Profile and Histology in Rats with Mild Cognitive Impairment (MCI) induced by AlCl3: Study Protocol

PLOS One

Dear Dr. Joseph,

Thank you for submitting your manuscript to PLOS ONE. After careful consideration, we have decided that your manuscript does not meet our criteria for publication and must therefore be rejected.

Although the manuscript is presented as a study protocol, large sections of the text are written in the style of a research paper, with outcome-oriented statements and speculative conclusions about expected synergistic effects. At the same time, no actual histological or biochemical results are presented, which creates confusion about the nature and purpose of the manuscript. A protocol should clearly focus on the study design, rationale, and methodological details, rather than predicting specific outcomes. In addition, key presentation issues noted in previous rounds, such as the absence of clear experimental diagrams or timelines in the main manuscript, have not been adequately addressed. Given that these concerns persist after multiple rounds of revision, the manuscript does not meet the journal’s standards in its current form, and we must therefore decline it.

I am sorry that we cannot be more positive on this occasion, but hope that you appreciate the reasons for this decision.

Kind regards,

Hira Rafi

Academic Editor

PLOS One

[Note: HTML markup is below. Please do not edit.]

Reviewers' comments:

Reviewer's Responses to Questions

**Comments to the Author**

1. Does the manuscript provide a valid rationale for the proposed study, with clearly identified and justified research questions?

The research question outlined is expected to address a valid academic problem or topic and contribute to the base of knowledge in the field.

Reviewer #1: Yes

Reviewer #4: Yes

**********

2. Is the protocol technically sound and planned in a manner that will lead to a meaningful outcome and allow testing the stated hypotheses?

The manuscript should describe the methods in sufficient detail to prevent undisclosed flexibility in the experimental procedure or analysis pipeline, including sufficient outcome-neutral conditions (e.g. necessary controls, absence of floor or ceiling effects) to test the proposed hypotheses and a statistical power analysis where applicable. As there may be aspects of the methodology and analysis which can only be refined once the work is undertaken, authors should outline potential assumptions and explicitly describe what aspects of the proposed analyses, if any, are exploratory.

Reviewer #1: Yes

Reviewer #4: Yes

**********

3. Is the methodology feasible and described in sufficient detail to allow the work to be replicable?

Descriptions of methods and materials in the protocol should be reported in sufficient detail for another researcher to reproduce all experiments and analyses. The protocol should describe the appropriate controls, sample size calculations, and replication needed to ensure that the data are robust and reproducible.

Reviewer #1: Yes

Reviewer #4: Yes

**********

4. Have the authors described where all data underlying the findings will be made available when the study is complete?

The PLOS Data policy requires authors to make all data underlying the findings described in their manuscript fully available without restriction, with rare exception, at the time of publication. The data should be provided as part of the manuscript or its supporting information, or deposited to a public repository. For example, in addition to summary statistics, the data points behind means, medians and variance measures should be available. If there are restrictions on publicly sharing data—e.g. participant privacy or use of data from a third party—those must be specified.

Reviewer #1: Yes

Reviewer #4: Yes

**********

5. Is the manuscript presented in an intelligible fashion and written in standard English?

PLOS ONE does not copyedit accepted manuscripts, so the language in submitted articles must be clear, correct, and unambiguous. Any typographical or grammatical errors should be corrected at revision, so please note any specific errors here.

Reviewer #1: Yes

Reviewer #4: Yes

**********

6. Review Comments to the Author

Please use the space provided to explain your answers to the questions above and, if applicable, provide comments about issues authors must address before this protocol can be accepted for publication. You may also include additional comments for the author, including concerns about research or publication ethics.

You may also provide optional suggestions and comments to authors that they might find helpful in planning their study.

(Please upload your review as an attachment if it exceeds 20,000 characters)

Reviewer #1: Publishable subject to minor corrections in formatting and grammar. The article in the present form is publishable and adds value to the subject.

Reviewer #4: I am satisfied with the changes the authors made in the revision which improves the manuscript. Therefore, I recommend this manuscript for publication.

**********

7. PLOS authors have the option to publish the peer review history of their article (what does this mean?). If published, this will include your full peer review and any attached files.

If you choose “no”, your identity will remain anonymous but your review may still be made public.

**Do you want your identity to be public for this peer review?**  For information about this choice, including consent withdrawal, please see our Privacy Policy.

Reviewer #1: **Yes:**  Dr Raja Chakraverty

Reviewer #4: No

**********

[NOTE: If reviewer comments were submitted as an attachment file, they will be attached to this email and accessible via the submission site. Please log into your account, locate the manuscript record, and check for the action link "View Attachments". If this link does not appear, there are no attachment files.]

- - - - -

For journal use only: PONEDEC3

# Author comment: Author response to Decision Letter 3

**DOI:** 10.1371/journal.pone.0349933.r006

15 Apr 2026

Comment

1. Although the manuscript is presented as a study protocol, large sections of the text are written in the style of a research paper, with outcome-oriented statements and speculative conclusions about expected synergistic effects.

Response

We appreciate the reviewer’s observation. The excerpts highlighted from the Introduction and Discussion sections were not intended to present definitive or speculative findings but rather to outline the scientific rationale and hypotheses that guide the proposed study. In response to the comment, we have revised these paragraphs to remove wording that may resemble outcome-oriented statements. The revised text now clearly presents hypotheses and anticipated directions strictly within the context of existing literature, without implying predetermined results. Our intention was solely to articulate the theoretical basis for the study and to justify why the investigation is warranted, which is consistent with standard requirements for protocol manuscripts.

Comment

2. At the same time, no actual histological or biochemical results are presented, which creates confusion about the nature and purpose of the manuscript.

Response

We thank the reviewer for this clarification. As this submission is a study protocol, no histological, biochemical, or behavioral results are presented because data collection has not yet begun. The manuscript aims to describe the study design, planned analyses, methodological framework, and justification, in accordance with protocol-paper guidelines. We have now added a sentence in the Methods section to explicitly state that data collection is forthcoming to avoid any ambiguity regarding the manuscript type.

Comment

3. A protocol should clearly focus on the study design, rationale, and methodological details, rather than predicting specific outcomes.

Response

We fully agree with the reviewer. The manuscript indeed emphasizes study design, rationale, methodology, sample size justification, and planned analyses. Any statements that may have been misinterpreted as predicted outcomes were meant to serve only as hypotheses grounded in prior evidence. No definitive or speculative outcomes are provided.

Comment

4. In addition, key presentation issues noted in previous rounds, such as the absence of clear experimental diagrams or timelines in the main manuscript, have not been adequately addressed.

Response

We appreciate this comment. In the previous revision rounds, we addressed all reviewer feedback to the best of our understanding, including incorporating an experimental workflow and study timeline. Please find the response to the reviewers in the attached documents.

A document detailing the response to the reviewers and editor has also been attached.

Attachment Submitted filename: Response to Editor & Reviewers.docx

# Editor report: Decision Letter 3

**DOI:** 10.1371/journal.pone.0349933.r007

**Authors:** Vara Prasad Saka, John Falode, Hira Rafi, Tanja Grubić Kezele

8 May 2026

Effect of Cosmos caudatus Supplementation and Aerobic Exercise on Selected Neurobehavior, Biochemical Profile and Histology in Rats with Mild Cognitive Impairment (MCI) induced by AlCl3: Study Protocol

PONE-D-24-27017R3

Dear Dr. Joseph,

We’re pleased to inform you that your manuscript has been judged scientifically suitable for publication and will be formally accepted for publication once it meets all outstanding technical requirements.

Within one week, you’ll receive an e-mail detailing the required amendments. When these have been addressed, you’ll receive a formal acceptance letter and your manuscript will be scheduled for publication.

An invoice will be generated when your article is formally accepted. Please note, if your institution has a publishing partnership with PLOS and your article meets the relevant criteria, all or part of your publication costs will be covered. Please make sure your user information is up-to-date by logging into Editorial Manager at Editorial Manager® and clicking the ‘Update My Information' link at the top of the page. For questions related to billing, please contact billing support.

If your institution or institutions have a press office, please notify them about your upcoming paper to help maximize its impact. If they’ll be preparing press materials, please inform our press team as soon as possible -- no later than 48 hours after receiving the formal acceptance. Your manuscript will remain under strict press embargo until 2 pm Eastern Time on the date of publication. For more information, please contact onepress@plos.org.

Kind regards,

Tanja Grubić Kezele, Ph.D., M.D.

Academic Editor

PLOS One

Additional Editor Comments (optional):

Reviewers' comments:

# Editor report: Acceptance letter

**DOI:** 10.1371/journal.pone.0349933.r008

**Authors:** Vara Prasad Saka, John Falode, Hira Rafi, Tanja Grubić Kezele

PONE-D-24-27017R3

PLOS One

Dear Dr. Joseph,

I'm pleased to inform you that your manuscript has been deemed suitable for publication in PLOS One. Congratulations! Your manuscript is now being handed over to our production team.

At this stage, our production department will prepare your paper for publication. This includes ensuring the following:

* All references, tables, and figures are properly cited

* All relevant supporting information is included in the manuscript submission,

* There are no issues that prevent the paper from being properly typeset

You will receive further instructions from the production team, including instructions on how to review your proof when it is ready. Please keep in mind that we are working through a large volume of accepted articles, so please give us a few days to review your paper and let you know the next and final steps.

Lastly, if your institution or institutions have a press office, please let them know about your upcoming paper now to help maximize its impact. If they'll be preparing press materials, please inform our press team within the next 48 hours. Your manuscript will remain under strict press embargo until 2 pm Eastern Time on the date of publication. For more information, please contact onepress@plos.org.

You will receive an invoice from PLOS for your publication fee after your manuscript has reached the completed accept phase. If you receive an email requesting payment before acceptance or for any other service, this may be a phishing scheme. Learn how to identify phishing emails and protect your accounts at https://explore.plos.org/phishing.

If we can help with anything else, please email us at customercare@plos.org.

Thank you for submitting your work to PLOS ONE and supporting open access.

Kind regards,

PLOS ONE Editorial Office Staff

on behalf of

Prof. dr. Tanja Grubić Kezele

Academic Editor

PLOS One
